# LIM homeobox 2 promotes interaction between human iPS-derived hepatic progenitors and iPS-derived hepatic stellate-like cells

**DOI:** 10.1038/s41598-018-37430-9

**Published:** 2019-02-14

**Authors:** Masato Miyoshi, Sei Kakinuma, Akihide Kamiya, Tomoyuki Tsunoda, Jun Tsuchiya, Ayako Sato, Shun Kaneko, Sayuri Nitta, Fukiko Kawai-Kitahata, Miyako Murakawa, Yasuhiro Itsui, Mina Nakagawa, Seishin Azuma, Hiromitsu Nakauchi, Yasuhiro Asahina, Mamoru Watanabe

**Affiliations:** 1 Department of Gastroenterology and Hepatology, Tokyo Medical and Dental University (TMDU), Tokyo, Japan; 2 Department of Liver Disease Control, Tokyo Medical and Dental University (TMDU), Tokyo, Japan; 3 Department of Molecular Life Sciences, Tokai University School of Medicine, Isehara, Kanagawa, Japan; 4 Institute for Stem Cell Biology and Regenerative Medicine, Stanford University School of Medicine, Stanford, CA USA; 5 Division of Stem Cell Therapy, Institute of Medical Science, The University of Tokyo, Tokyo, Japan

## Abstract

Human induced pluripotent stem (iPS) cells can differentiate into hepatocyte lineages, although the phenotype of the differentiated cells is immature compared to adult hepatocytes. Improvement of cell-cell interactions between epithelium and mesenchyme is a potential approach to address this phenotype issue. In this study, we developed a model system for improving interactions between human iPS-derived hepatic progenitor cells (iPS-HPCs) and human iPS-derived hepatic stellate cell-like cells (iPS-HSCs). The phenotype of iPS-HSCs, including gene and protein expression profiles and vitamin A storage, resembled that of hepatic stellate cells. Direct co-culture of iPS-HSCs with iPS-HPCs significantly improved hepatocytic maturation in iPS-HPCs, such as their capacity for albumin production. Next, we generated iPS cell lines overexpressing LIM homeobox 2 (LHX2), which suppresses myofibroblastic changes in HSCs in mice. Hepatocytic maturation in iPS-HPCs was significantly increased in direct co-culture with iPS-HSCs overexpressing LHX2, but not in co-culture with a human hepatic stellate cell line (LX-2) overexpressing LHX2. LHX2 regulated the expression of extracellular matrices, such as laminin and collagen, in iPS-HSCs. In conclusion, this study provides an evidence that LHX2 upregulation in iPS-HSCs promotes hepatocytic maturation of iPS-HPCs, and indicates that genetically modified iPS-HSCs will be of value for research into cell-cell interactions.

## Introduction

Human induced pluripotent stem (iPS) cells are somatic cells that have been genetically reprogrammed to be pluripotent by the transient expression of genes essential for maintaining the properties of embryonic stem cells^1^. Human iPS cells and embryonic stem cells exhibit the potential for differentiation into hepatocyte lineages^2–4^. Utilization of human iPS-derived hepatocyte-like (iPS-Hep) cells as a genetic disease model^5^, viral infection model^6^, for drug screening, and in regenerative medicine^7^ has several substantial advantages compared with primary hepatocytes, such as the potential for unlimited expansion. Moreover, iPS-Hep cells with genetic modifications may be of value for research into various diseases. Our recent studies showed that iPS-Hep cells and iPS-derived hepatic progenitor cells (iPS-HPCs) are susceptible to the hepatitis B virus^6,8^. Previous studies also showed that the phenotypes of iPS-Hep cells are immature compared to adult hepatocytes with respect to albumin production, activity of cytochrome P450, and metabolic functions^9^. This problem of the immature nature of iPS-Hep cells as hepatocytes needs to be resolved.

During liver development, cell-cell interactions between foregut endodermal cells and endothelial cells play an essential role in hepatic specification^10^. Maturation of hepatocytes is also induced by a cell-cell interaction between hepatoblasts and septum transversum mesenchyme (STM) or hepatic stellate cells (HSCs)^11,12^. Consistent with this developmental process, co-culture of iPS-derived hepatic cells, mesenchymal stem cells, and human umbilical cord endothelial cells induces hepatic maturation of iPS-derived hepatic cells (termed iPS-liver bud)^7^. It is possible that co-culture of iPS-derived hepatic cells and iPS-derived hepatic stellate cell-like cells (iPS-HSCs) contributes to hepatic maturation^13–15^.

HSCs are derived from MESP1^+^ mesoderm, STM, and mesothelium of liver during development^16,17^. HSCs retain a quiescent state, store vitamin A in the cytosol, assist the metabolic function of hepatocytes, and maintain extracellular matrices (ECM) *in vivo*^18^. Once HSCs are activated by inflammation signals, such as transforming growth factor (TGF)-β due to liver damage, they transdifferentiate into myofibroblasts, lose the vitamin A droplet, and actively produce ECM. Thus, activated HSCs play a central role in liver fibrosis. In contrast to the *in vivo* phenotype, it is difficult to maintain the quiescent phenotype of HSCs *in vitro*, because primary HSCs immediately transdifferentiate into myofibroblasts^18^. Recent reports have shown that several molecules play a role in the maintenance of HSC quiescence. Reduction of peroxisome proliferator-activated receptor gamma (*PPARγ*) expression is associated with the activation of HSCs^19^, and restoration of PPARγ reverses the activated HSCs to the quiescent phenotype^20^. However, the molecular mechanisms that regulate HSC quiescence are not fully elucidated.

A previous study showed that the transcription factor, LIM homeobox 2 (LHX2), may be important for the inhibition of HSC activation. LHX2 carries a LIM domain, a cysteine-rich zinc-binding domain^21^. In *Lhx2*-deficient mice, formation of STM is not impaired in fetal livers, whereas the mice develop severe hepatic fibrosis during fetal development due to myofibroblastic changes in liver mesenchymal cells. This suggests that Lhx2 is dispensable for HSC development in the fetus but is important for the maintenance of the quiescent phenotype of HSCs in mice^22^. Lhx2 also regulates neural development^23,24^ and homeostasis in hair follicle stem cells in mice^25^. LHX2 promotes hematopoietic differentiation of human iPS cells^26,27^. However, the function of LHX2 in human HSCs and human iPS-HSCs is unclear. Additionally, it is not known whether upregulation of LHX2 in human HSCs contributes to maturation of iPS-derived hepatic cells.

The aims of this study were to determine whether interactions between iPS-HSCs and iPS-derived hepatic cells contribute to increased maturation of iPS-derived hepatic cells, and to evaluate the effects of induced LHX2 upregulation on iPS-HSC functions. Here, we demonstrated that the phenotype of human iPS-HSCs resembled that of HSCs, and that co-culture of iPS-HPCs with iPS-HSCs induced hepatic maturation in iPS-HPCs. Upregulation of LHX2 in iPS-HSCs increased hepatic maturation compared with normal iPS-HSCs. LHX2 upregulation altered the ECM-expression profile in the iPS-HSCs, which is different from that in a human HSC cell line, LX-2.

## Results

### Differentiation of human iPS cells into mesenchymal cells exhibiting a hepatic stellate cell (iPS-HSC) phenotype

**Figure 1:**
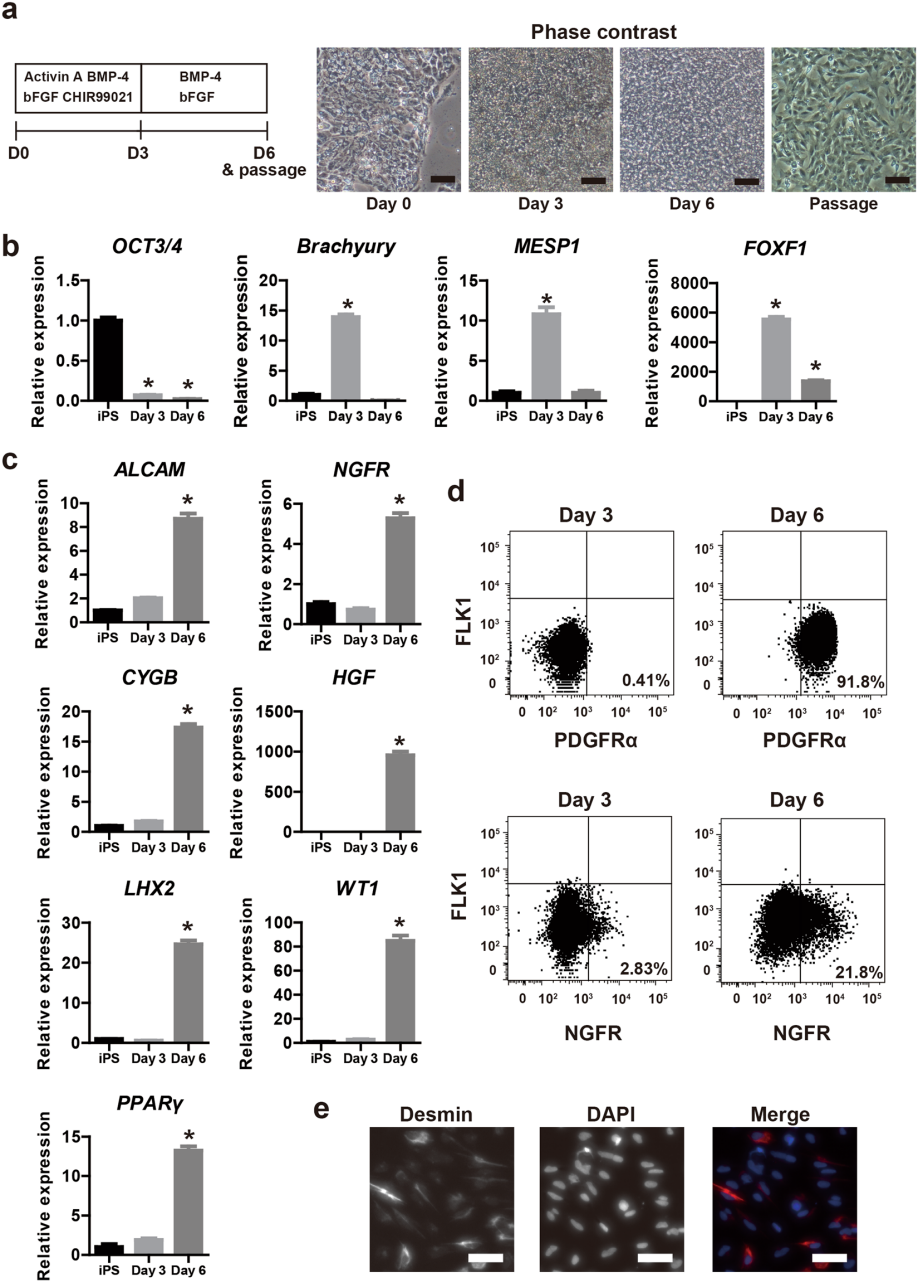
Differentiation of hepatic stellate cell-like cells derived from human induced pluripotent stem cells (iPS-HSCs). (**a**) Left panel: protocol for iPS-HSC differentiation. Culture medium was supplemented with the following factors: Days 1–3, 10 ng/ml basic fibroblast growth factor (bFGF), 10 μM CHIR99021, 30 ng/ml bone morphogenetic protein-4 (BMP-4), and 10 ng/ml activin A; Days 4–6, 100 ng/ml bFGF and 50 mg/ml BMP-4. Right panel: phase contrast views showing cells at day 0, day 3, and day 6 before passage, and at day 6 after passage. Scale bars: 100 μm. (**b**) Expression of *OCT3/4*, *Brachyury*, mesoderm posterior basic helix-loop-helix transcription factor *1* (*MESP1*), and forkhead box F1 (*FOXF1*) in human iPS-derived cells at day 0 (iPS cells), day 3, and day 6. The y-axis represents the ratio of expression relative to the means of day 0 (iPS cells). (**c**) Expression of activated leukocyte cell adhesion molecule (*ALCAM*), nerve growth factor receptor (*NGFR*), cytoglobin (*CYGB*), hepatocyte growth factor (*HGF*), LIM homeobox 2 (*LHX2*), Wilms tumor 1 (*WT1*), and peroxisome proliferator-activated receptor gamma (*PPARγ*) in human iPS-derived cells, which are related to differentiation of septum transversum mesenchyme (STM) and hepatic stellate cells (HSCs). The y-axis represents the ratio of expression relative to means of day 0 (iPS cells). (**d**) Flow cytometric analysis of human iPS-derived cells at day 3 and day 6. The x- and y-axis in the upper panels shows platelet derived growth factor receptor alpha (PDGFRα) and fetal liver kinase 1 (FLK1), respectively. The x- and y-axis in the lower panels shows NGFR and FLK1, respectively. (**e**) Immunostaining of desmin (red). Nuclei were stained with 4′, 6-diamidine-2′-phenylindole dihydrochloride (DAPI, blue). Scale bars: 50 µm. Based on these data, human iPS-derived cells at day 3 and day 6 were termed iPS-mesodermal progenitor (iPS-MP) cells and iPS-HSCs, respectively. Results represent the mean ± SD of three separate experiments. *P < 0.05.

Hepatic stellate cells develop from mesoderm, STM and mesothelium^16,17^. To obtain iPS-derived mesenchymal cells exhibiting a hepatic stellate cell phenotype (iPS-derived hepatic stellate cell-like cells, iPS-HSCs), we modified previously described protocols for differentiation of human iPS cells into mesoderm or lateral plate mesoderm (LPM)^28–30^. The protocol for iPS-HSC differentiation is shown in Fig. 1a. In the “bABC protocol”, human iPS cells (RIKEN-2F) were differentiated into mesodermal progenitor cells (iPS-MP) by treatment with basic fibroblast growth factor (bFGF), activin A, bone morphologic protein-4 (BMP-4), and GSK-3β inhibitor (CHIR99021) for 3 days. The cells were then cultured with bFGF and BMP-4 from days 4 to 6. The cultured cells actively proliferated under this differentiation protocol, and exhibited a spindle shape similar to that of mesenchymal cells (Fig. 1a).

After differentiation for 3 days, NCAM^+^ and EpCAM^−^ cells constituted 76% of the iPS-derived cells, and most of these cells had differentiated into mesodermal progenitor cells (iPS-MP cells, Supplementary Fig. 1a)^28^. By comparison, the NCAM^+^ and EpCAM^−^ fraction among differentiated cells was 45% and 19% using the “bB protocol” (bFGF and CHIR99021 for the first 3 days) and “bC-bAB protocol” (bFGF and CHIR99021 for a day, and bFGF, activin A, and BMP-4 on days 2 and 3), respectively (Supplementary Fig. 1a). These data show the higher efficiency of the bABC protocol for inducing differentiation of human iPS cells into iPS-MP cells compared to the bB and bC-bAB protocols.

An expression analysis of differentiated (iPS-MP) cells was undertaken at days 0 to 3 for *Oct3/4* (a pluripotent marker), *Brachyury* (a marker of primitive streak), and mesoderm posterior basic helix-loop-helix transcription factor 1 (*MESP1*, a mesoderm marker). On day 3, expression of these genes was significantly decreased compared to peak expression level found at day 0 for *Oct3*/*4*, at day 1 for *Brachyury*, and at day 2 for *MESP1* (Supplementary Fig. 1b). By contrast, expression of forkhead box F1 (*FOXF1*, a LPM marker) significantly increased in iPS-MP cells using the bABC differentiation protocol (Supplementary Fig. 1b). These data indicated that the iPS-derived cells had differentiated into the LPM lineage. After differentiation for 6 days, *Oct3/4* expression in the iPS-derived cells markedly decreased, and expression of *Brachyury* and *MESP1* was significantly reduced compared to iPS-MP cells (Fig. 1b). *FOXF1* expression in iPS-derived cells at day 6 remained significantly higher than at day 0 (Fig. 1b).

Next, the expression of HSC marker genes in iPS-derived cells was assessed. Expression of activated leukocyte cell adhesion molecule (*ALCAM*, a mesothelial cell marker), nerve growth factor receptor (*NGFR*, a typical HSC marker), Wilms tumor 1 (*WT1*, a transcription factor related to HSCs), *PPARγ*, and *LHX2* were significantly increased in iPS-derived cells on day 6 compared to day 0 (Fig. 1c). Hepatocyte growth factor (*HGF*) and cytoglobin (*CYGB*) were also up-regulated in iPS-derived cells on day 6 (Fig. 1c). A flow cytometric analysis demonstrated that most iPS-derived cells on day 6 produced platelet-derived growth factor receptor (PDGFRα, a marker for the mesenchymal cell lineage) and that ~20% of cells produced NGFR; the PDGFRα^+^ or NGFR^+^ fraction among iPS-MP cells was very low (Fig. 1d). An immunostaining analysis showed that iPS-derived cells on day 6 produced desmin (a HSC marker protein, Fig. 1e). Thus, we term the day 6 iPS-derived cells as “iPS-HSCs”.

To evaluate whether our protocol (Fig. 1a) can induce other iPS cell lines to differentiate into iPS-HSCs, we cultured the iPS cell line PB001 using the bABC protocol. Analyses of gene expression showed that day 6 PB001-derived cells had a similar profile to that of RIKEN-2F iPS-derived cells (Supplementary Fig. 1c). Thus, our protocol (Fig. 1a) was able to induce human iPS-HSC differentiation in other cell lines. Our data indicated that the differentiated iPS-derived cells are mesodermal lineage cells that exhibit a phenotype consistent with that of HSCs.

### Vitamin A storage and myofibroblastic change in iPS-HSCs

Human HSCs display characteristic features of vitamin A storage and myofibroblastic change (HSC-activation). To confirm that human iPS-HSCs have the same phenotype as human HSCs, we evaluated these features in iPS-HSCs.

**Figure 2:**
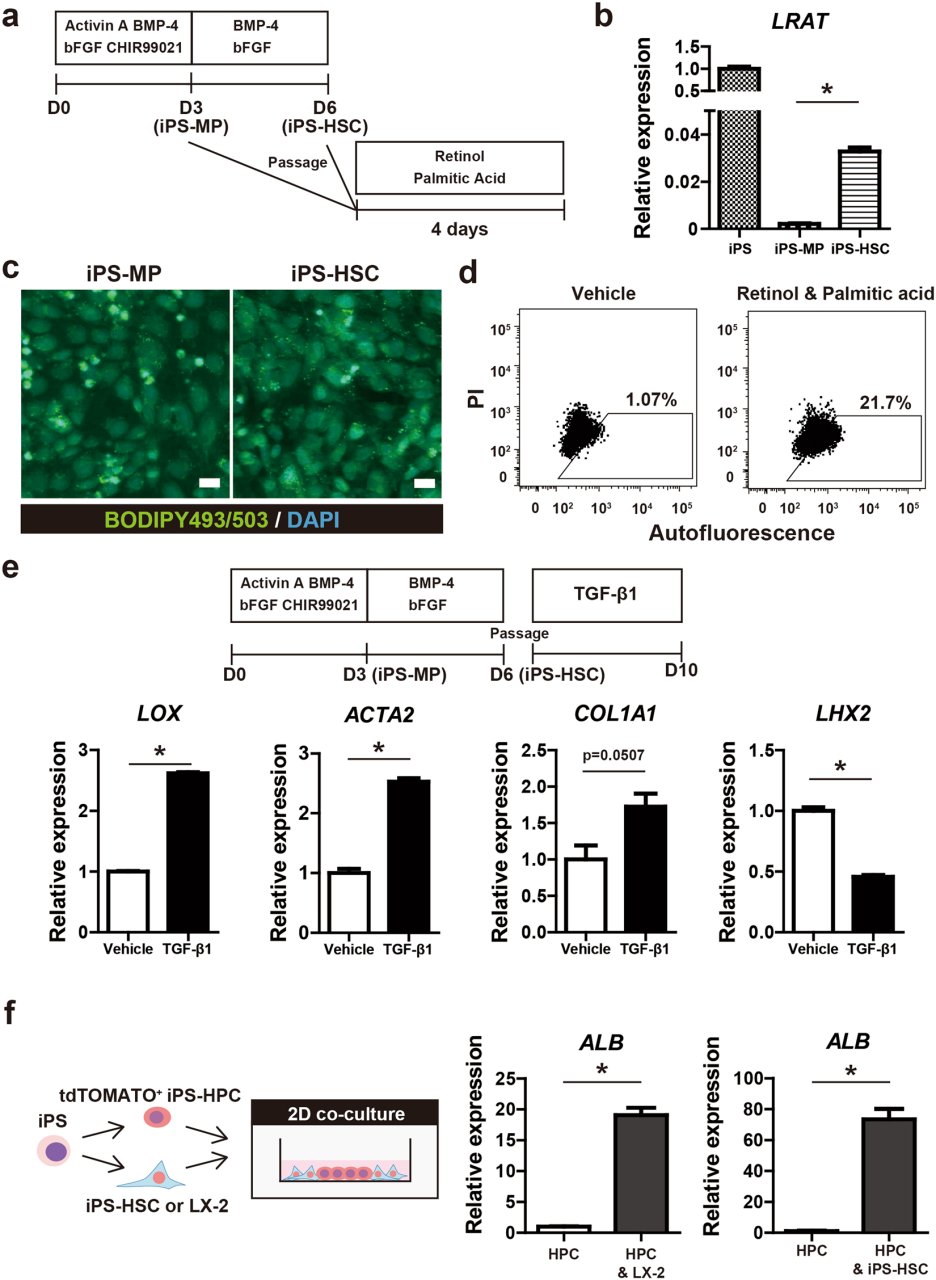
Analysis on the phenotypes of iPS-HSCs related to human HSCs. (**a**) Protocol for the analysis of vitamin A storage in iPS-derived cells cultured as described in Fig. 1a. iPS-MP cells (at day 3) and iPS-HSCs (at day 6) were incubated with 5 μM retinol and 100 μM palmitic acid for 4 days. (**b**) Expression of lecithin retinol acyltransferase (*LRAT*) in iPS cells, iPS-MP cells, and iPS-HSCs. The y-axis represents the ratio of expression relative to means of iPS cells. (**c**) Fluorescent images of lipid droplets in iPS-derived cells incubated with retinol and palmitic acid. The cells were stained with BODIPY495/503 (green), and nuclei were stained with DAPI (blue). Positive signals were evident in iPS-HSCs compared to those in iPS-MP cells. Scale bars: 10 μm. (**d**) Flow cytometric analysis of autofluorescence in iPS-HSCs cultured with vehicle (left) or retinol plus palmitic acid (right). High autofluorescence was observed in ~20% of iPS-HSCs. (**e**) Upper panel shows the protocol for the experiment of TGF-β1 stimulation. Lower panel shows expression of genes related to HSC activation: lysyl oxidase (*LOX*), α-smooth muscle actin (*ACTA2*), type I collagen alpha 1 chain (*COL1A1*) and *LHX2*. The y-axis represents the ratio of expression relative to the means of vehicle-treated cells. (**f**) Left panel: schema for the 2-dimensional (2D) direct co-culture of tdTOMATO^+^ human iPS-derived hepatic progenitor cells (iPS-HPCs) and iPS-HSCs using the Cytoselect system. Right panel: Expression of albumin (*ALB*) in iPS-HPCs and in iPS-HPCs co-cultured with iPS-HSCs or LX-2 cells (a human hepatic stellate cell line). The y-axis represents the ratio of expression relative to the means of iPS-HPCs. Results represent the mean ± SD of three separate experiments. *P < 0.05.

To investigate the ability of iPS-HSCs to store vitamin A, iPS-MP cells (at day 3) and iPS-HSCs (at day 6) were incubated with retinol and palmitic acid for 4 days (Fig. 2a). Expression of lecithin retinol acyltransferase (*LRAT*, a molecule related to vitamin A metabolism and known as a marker of quiescence in HSCs) was significantly increased in iPS-HSCs compared with iPS-MP cells (Fig. 2b). Lipid droplets in iPS-HSCs after incubation with retinol and palmitic acid were detected, whereas they were infrequent in iPS-MP cells (Fig. 2c). A flow cytometric analysis showed that ~20% of iPS-HSCs had increased autofluorescence after incubation with retinol and palmitic acid compared to control cells treated with vehicle (Fig. 2d), indicating that iPS-HSCs can store vitamin A in the cytoplasm.

To investigate whether iPS-HSCs are activated by inflammatory stimulation and are transformed into myofibroblast-like cells, we examined the response of iPS-HPCs to TGF-β stimulation as this is known to activate HSCs (Fig. 2e)^18^. In TGF-β1 treated cells, the expression of lysyl oxidase (*LOX*), type I collagen alpha 1 chain (*COL1A1*), and α-smooth muscle actin (*ACTA2*) in was significantly increased compared to vehicle-treated cells (Fig. 2e). By contrast, the expression of *LHX2* was significantly decreased after TGF-β1 stimulation (Fig. 2e). These results demonstrated that the phenotype of iPS-HSCs, with regard to vitamin A storage and myofibroblastic change, resembles that of human HSCs.

### iPS-HSCs promote hepatic maturation of iPS-HPCs in co-culture

To investigate whether iPS-HSCs promoted hepatic maturation of iPS-HPCs, we co-cultured iPS-HSCs with iPS-HPCs in transwell or 2-dimensional (2D) direct cultures. In transwell co-culture, expression of α-fetoprotein (*AFP*) and albumin (*ALB*) was unchanged in iPS-HPCs co-cultured with either iPS-HSCs or the human hepatic stellate cell line LX-2 compared to cells not in co-cultures (Supplementary Fig. 2). In 2D direct co-cultures, *ALB* expression was significantly increased in iPS-HPCs co-cultured with iPS-HSCs or LX-2 cells (Fig. 2f). These data indicated that iPS-HSCs can induce hepatic maturation in iPS-HPCs, and that the cell-cell interaction effect is not due to humoral factors secreted by the iPS-HSCs.

### Generation of doxycycline (Dox)-inducible LHX2-overexpressing human iPS cell lines and differentiation into iPS-HSCs

**Figure 3:**
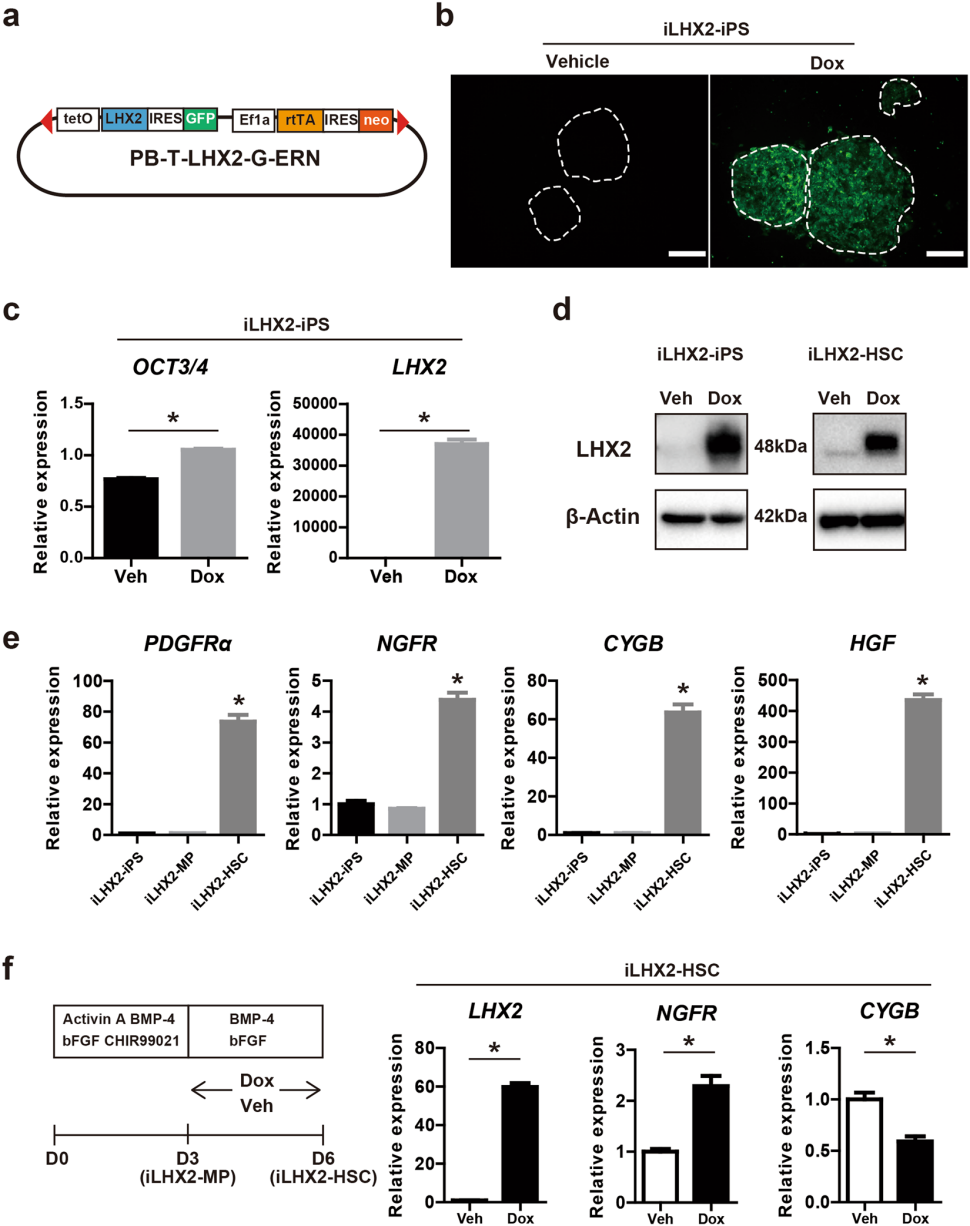
Generation of iPS cell lines doxycycline-inducible overexpressing LHX2 (iLHX2) and differentiation of iLHX2 into iPS-HSCs. (**a**) Schema for construct of a self-contained, drug-inducible expression vector based on the PiggyBac transposon (PB-T-LHX2-G-ERN) used in this study. Tetracycline resistant operon (tetO), LHX2 gene, internal ribosomal entry site (IRES), green fluorescence protein (GFP), EF1α promoter sequences (EF1α), reverse tetracycline transactivator gene (rtTA), and neomycin-resistant gene (neo) are shown. (**b**) Representative view of pooled iPS cell clones containing genomic transposon integrations of LHX2 (iLHX2-iPS). Activation of LHX2 expression in response to Dox was indirectly monitored by co-incident GFP (green). Scale bars: 200 μm. (**c**) Expression of *OCT3/4* and *LHX2* in iLHX2-iPS clones cultured with vehicle (Veh) or doxycycline (Dox). The y-axis represents the ratio of expression relative to the means of iPS cells. (**d**) Immunoblot analysis of iLHX2-iPS clones and iPS-HSCs derived from iLHX2-iPS clones (iLHX2-HSC), which were cultured with Veh or Dox. (**e**) Expression of *PDGFRα*, *NGFR*, *CYGB*, and *HGF* in iLHX2-iPS clones, iPS-MP derived from iLHX2-iPS clones (iLHX2-MP), and iLHX2-HSCs, which were cultured without Dox. (**f**) Left panel: protocol for iLHX2-HSCs cultured with Dox at days 4–6. Expression of *LHX2*, *NGFR*, and *CYGB* in iLHX2-HSCs cultured with Veh or Dox. Results represent the mean ± SD of three separate experiments. *P < 0.05.

Next, we examined the ability of the transcription factor LHX2 to enhance the iPS-HSC-induced hepatic maturation of iPS-HPCs. *LHX2* expression was increased in iPS-HSCs compared to iPS cells and iPS-MP cells (Fig. 1b); however, expression in iPS-HSCs was lower than in the HSC cell line LX-2 (Supplementary Fig. 3a). Thus, we generated human iPS cell lines overexpressing LHX2. We constructed a self-contained, tetracycline-inducible expression vector based on the PiggyBac transposon as previously described (Fig. 3a)^6,31^. Activation of gene expression in response to Dox was indirectly monitored by coincident green fluorescent protein (GFP) activation (Fig. 3b). We generated and selected >10 different iPS cell lines overexpressing LHX2 (and termed these iLHX2-iPS cells); in these cells, *LHX2* expression in undifferentiated iPS cells was induced by Dox (Fig. 3c,d). *Oct3/4* expression was slightly increased in Dox-treated iLHX2-iPS cells (Fig. 3c), consistent with a previous report that LHX2 interferes with an initial step of iPS cell differentiation^32^. Immunoblot analysis confirmed that the production of LHX2 protein was up-regulated in Dox-treated iLHX2 cells (Fig. 3d).

iLHX2-iPS cells were cultured using the protocol for iPS-HSC differentiation (shown in Fig. 1a) without Dox to determine whether they could differentiate into HSC-lineage cells. After differentiation, expression of *NGFR*, *CYGB*, *HGF* was significantly increased compared to iLHX2-iPS cells. This result indicated that iLHX2-iPS cells differentiated into HSC-lineage cells in a similar manner to normal iPS cells using this culture protocol; we termed these differentiated iLHX2 cells as iLHX2-HSCs (Fig. 3e). Next, to determine whether LHX2 overexpression affects iPS-HSC differentiation, iLHX2-iPS cells were differentiated and treated with Dox from day 4 to 6 (Fig. 3f). Expression of *LHX2* in Dox-treated iLHX2-HSCs was ~60 times higher than in vehicle-treated cells (Fig. 3f). Immunoblots showed that the LHX2 protein was increased in Dox-treated iLHX2-HSCs compared with vehicle-treated cells (Fig. 3d). *NGFR* expression in Dox-treated iLHX2-HSCs was significantly increased compared to vehicle-treated cells, however *CYGB* expression was decreased by LHX2 overexpression (Fig. 3f). These data indicated that LHX2 overexpression in differentiating iPS cells has no influence on iPS-HSC differentiation.

### LHX2 overexpression in iPS-HSCs facilitates hepatic maturation of iPS-HPCs in co-culture

**Figure 4:**
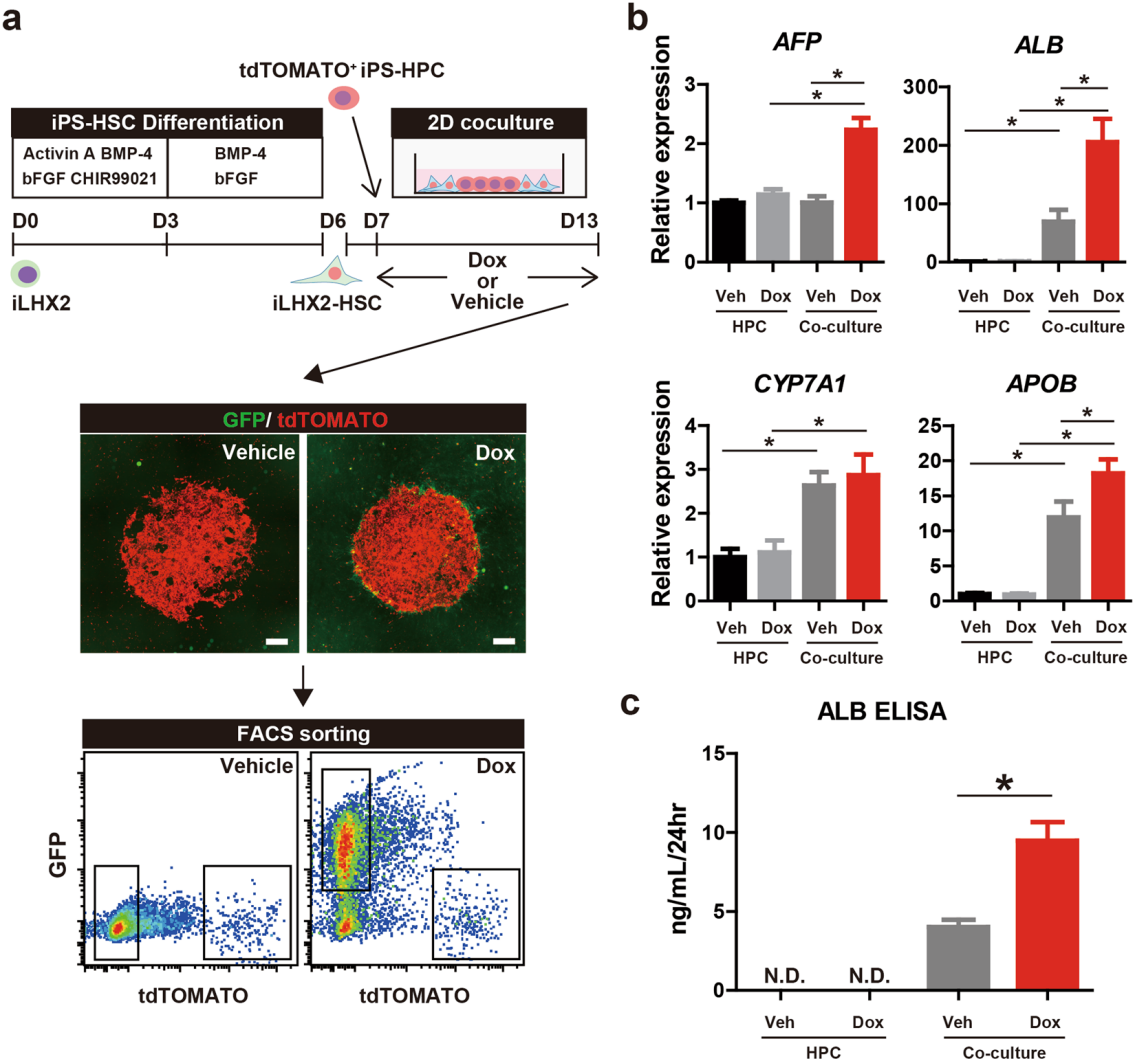
Overexpression of LHX2 in iPS-HSCs facilitates hepatic maturation of iPS-HPCs. (**a**) Upper panel: schema for 2D direct co-culture of tdTOMATO^+^ iPS-HPCs (red) and iLHX2-HSCs (green) using the Cytoselect system. Middle panel: representative images of co-cultured cells. Scale bars: 500 μm. Lower panel: representative density plots from flow cytometric analysis of co-cultured cells with vehicle (Veh) or Dox. Gated fractions of co-cultures cells were sorted and analyzed. (**b**) Expression of α-fetoprotein (*AFP*), *ALB*, cytochrome P450, family 7, subfamily A, polypeptide 1 (*CYP7A1*), and apolipoprotein B (*APOB*) in iPS-HPCs. The y-axis represents the ratio of expression relative to the mean of iPS-HPCs cultured without iPS-HSCs and Dox. (**c**) Enzyme-linked immunosorbent assay (ELISA) of ALB. ALB production in iPS-HPCs was significantly increased by overexpression of LHX2 in iPS-HSCs. Results represent the mean ± SD of three separate experiments. *P < 0.05.

Functional changes in iPS-HSCs due to LHX2 overexpression were analyzed in 2D direct co-cultures of iLHX2-HSCs and iPS-HPCs. After co-culture for 6 days, cells were harvested and sorted using FACS; tdTOMATO-labelled iPS-HPCs and GFP-labelled iLHX2-HSCs were collected (Fig. 4a). An expression analysis showed that *AFP* expression was significantly increased in iPS-HPCs co-cultured with Dox-treated iLHX2-HSCs compared to other groups (Fig. 4b). Expression and production of ALB was significantly increased in iPS-HPCs co-cultured with vehicle-treated iLHX2-HSCs compared to cells not subjected to co-culture. ALB expression and production was also significantly increased in iPS-HPCs co-cultured with Dox-treated iLHX2-HSCs compared to vehicle-treated iLHX2-HSCs (Fig. 4b,c). These data clearly indicated that LHX2 expression in iPS-HSCs facilitated ALB production in iPS-HPCs in co-culture. Expression of apolipoprotein B (*ApoB*) was significantly increased in iPS-HPCs co-cultured with Dox-treated iLHX2-HSCs compared to vehicle-treated iLHX2-HSCs (Fig. 4b). *CYP7A1* expression in HPCs co-cultured with Dox-treated or vehicle-treated iLHX2-HSCs was increased (Fig. 4b). These data indicated that LHX2 expression in iPS-HSCs facilitated hepatic maturation of iPS-HPCs via direct cell-cell interactions.

### LHX2 overexpression in LX-2 cells does not facilitate hepatic maturation of iPS-HPCs in co-culture

To determine whether LHX2 overexpression in the HSC cell line LX-2 induced hepatic maturation of iPS-HPCs to the same extent as iPS-HSCs, we constructed LX-2 cell lines overexpressing LHX2 (LX-2-LHX2) as described for iPS cells (Supplementary Fig. 3b). A previous study showed that Lhx2 overexpression in murine HSCs cells does not contribute to maintenance of the quiescent phenotype^33^. Expression analysis showed that expression of *ACTA2* was decreased in Dox-treated LX-2-LHX2 cells; however, expression of *COL1A1* in these cells was similar to that in vehicle-treated LX-2-LHX2 cells and expression of *LOX* was slightly increased (Supplementary Fig. 3c). There was no difference in the numbers of desmin-producing cells between vehicle- and Dox-treated LX-2-LHX2 (Supplementary Fig. 3d). These results indicated that LX-2 cells overexpressing LHX2 did not switch to a quiescent phenotype. Overexpression of LHX2 in LX-2 cells did not change expression of *AFP*, *ALB*, *CYP7A1*, and *ApoB* in iPS-HPCs in both transwell and 2D direct co-culture (Supplementary Fig. 3e,f). Down-regulation of LHX2 in LX-2 cells did not change expression of *AFP* and *ALB* in iPS-HPCs in 2D direct co-culture (Supplementary Fig. 3g,h). These data indicated that the change of LHX2 expression in LX-2 cells has no effect on hepatic maturation of iPS-HPCs in direct co-culture, in contrast to that in iPS-HSCs.

### Change of ECM expression profile in LHX2-overexpressed iPS-HSCs

The mechanism through which overexpression of LHX2 in iPS-HSCs facilitates hepatic maturation of iPS-HPCs was investigated using a microarray assay. Vehicle-treated or Dox-treated iLHX2-HSCs were co-cultured with iPS-HPCs prior to analysis. A gene ontogeny analysis showed that LHX2 overexpression up-regulated genes related to development of the neural crest that differentiates into HSCs in zebrafish^34^. Genes related to development of neurons and brain were also up-regulated by LHX2 overexpression (Supplementary Fig. 4a)^23,35^. These results confirm that the overexpressed LHX2 in iLHX2-HSCs functioned as a transcription factor against LHX2-target genes. The microarray assay also showed that genes related to cell adhesion and collagen fibril organization were down-regulated by LHX2 overexpression; these down-regulated genes were associated with ECM production (Supplementary Fig. 4b).

**Figure 5:**
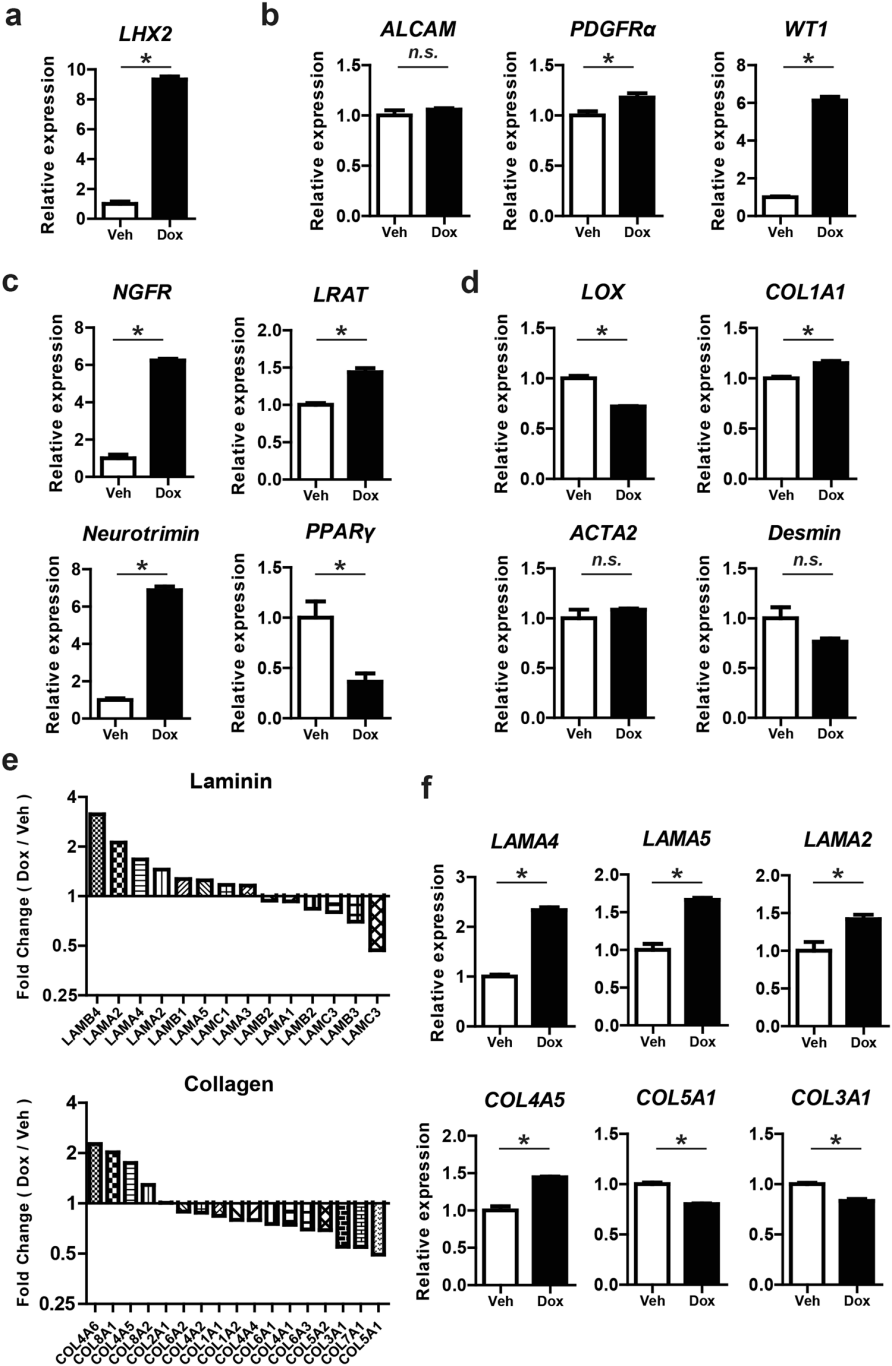
LHX2 overexpression in iPS-HSCs changes the expression profile of the extracellular matrix (ECM), and induces gene expression related to the quiescence phenotype of HSCs. (**a)** Expression of *LHX2* in Veh- or Dox-treated iLHX2-HSCs co-cultured with iPS-HPCs. (**b**,**c**) Expression of genes related to HSCs (b, *ALCAM*, *PDGFRα*, and *WT1*) and genes related to the quiescence phenotype of HSCs (c, *NGFR*, *LRAT, Neurotrimin*, and *PPARγ*) in Veh- or Dox-treated iLHX2-HSCs. (**d**) Expression of genes related to HSC activation (*LOX*, *ACTA2*, *COL1A1*, and *Desmin*) in Veh- or Dox-treated iLHX2-HSCs co-cultured with iPS-HPCs. (**e**) Expression profile of laminin and collagen based on microarray analysis. The y-axis represents the ratio of expression relative to Veh-treated iLHX2-HSCs co-cultured with iPS-HPCs. (**f**) Quantitative RT-PCR analysis of laminin (*LAMA2*, *LAMA4*, and *LAMA5*) and collagen (*COL3A1*, *COL4A5*, and *COL5A1*) in iLHX2-HSCs co-cultured with iPS-HPCs. Results represent the mean ± SD of three separate experiments. The y-axis in (**a**–**d**) and (**f**) represents the ratio of expression relative to Veh-treated iLHX2-HSCs co-cultured with iPS-HPCs. *P < 0.05.

Expressions of *PDGFRα*, *WT1, NGFR*, *LRAT*, and *Neurotrimin* (a marker for quiescent HSCs)^36^ were significantly up-regulated in Dox-treated iLHX2-HSCs as well as that of *LHX2*, whereas the expressions of *ALCAM* and *PPARγ* were not changed by LHX2 overexpression (Fig. 5a–c). Expression of *LOX* was down-regulated and that of *COL1A1* was up-regulated in Dox-treated iLHX2-HSCs; expression of *ACTA2* and *Desmin* was not significantly changed (Fig. 5d). The numbers of desmin-producing cells in Dox-treated iLHX2-HSCs were equal to those in vehicle-treated cells (Supplementary Fig. 5a,b). Taken together, these results suggested that LHX2 overexpression in iPS-HSCs partially maintains the quiescence phenotype of HSCs.

The microarray analysis showed that humoral factors and expression of *FGF7* and *HGF* were increased in Dox-treated iLHX2-HSCs compared with vehicle-treated iLHX2-HSCs. A quantitative RT-PCR assay confirmed that expression of *FGF7* and *HGF* was up-regulated (Supplementary Fig. 5c). The function of such humoral factors in hepatic maturation of iPS-HPCs induced by Dox-treated iLHX2-HSCs was analyzed using iPS-HPCs cultured in media supplemented with HGF, FGF7, and HGF plus FGF7. Expression of *AFP* and *ALB* in iPS-HPCs did not change significantly after treatment with the humoral factors (Supplementary Fig. 5d). Furthermore, expression of *AFP* and *ALB* in iPS-HPCs did not change significantly in transwell co-cultures with Dox-treated iLHX2-HSCs (Supplementary Fig. 5e). These results showed that the humoral factors derived from Dox-treated iLHX2-HSCs were insufficient to induce hepatic maturation in iPS-HPCs.

The microarray analysis also demonstrated that expression of collagen and laminin (major components of the liver ECM) was clearly changed in Dox-treated iLHX2-HSCs compared with vehicle-treated iLHX2-HSCs (Fig. 5e). Expression of laminin-family genes was up-regulated by LHX2 overexpression, whereas expression of collagen-family genes, except for type IV collagen, was down-regulated. A quantitative RT-PCR analysis confirmed that expression of *LAMA4*, *LAMA2*, *LAMA5*, and *COL4A5* (type IV collagen) was significantly increased in Dox-treated iLHX2-HSCs compared to controls (Fig. 5f). Expression of *COL3A1* and *COL5A1* was significantly decreased in Dox-treated iLHX2-HSCs compared to controls (Fig. 5f). In contrast to iLHX2-HSCs, expressions of *COL3A1* and *COL5A1* were significantly increased in Dox-treated LX-2-LHX2 cells and *LAMA5* expression in such cells was significantly decreased relative to controls (Supplementary Fig. 6). These data showed that LHX2 overexpression in iLHX2-HSCs changed the expression profile of the ECM, and suggested that changes in the ECM were responsible for hepatic maturation of iPS-HPCs after co-culture with iLHX2-HSCs.

Previous studies showed that culture of iPS-derived cells on laminin-coated dishes supports their differentiation into the hepatic lineage and their maintenance as hepatoblast-like cells^37,38^. Thus, we analyzed expression of *AFP* and *ALB* in iPS-HPCs cultured on laminin-511-coated, type I collagen-coated, and fibronectin-coated dishes (the same dishes mentioned above in the 2D direct co-culture experiments). *AFP* expression in iPS-HPCs cultured on laminin-511-coated dishes was higher than on fibronectin-coated or type I collagen-coated dishes, whereas *ALB* expression in such cells was similar to that on fibronectin-coated or type I collagen-coated dishes (Supplementary Fig. 5d). These results indicated that the simple addition of a particular laminin is not enough to induce hepatic maturation in iPS-HPCs, and suggested that changes in the ECM expression profile, including production of various laminin and collagen proteins in LHX2-overexpressing iPS-HSCs, were responsible for the additive effects on hepatic maturation of iPS-HPCs.

## Discussion

This study demonstrated that LHX2 overexpression in iPS-HSCs promotes hepatic maturation in iPS-HPCs; this effect is mediated through modification of the expression profile of iPS-HSC-derived ECM and is not influenced by humoral factors (Figs 4 and 5). This is the first evidence that genetic modification of human hepatic non-parenchymal cells can facilitate hepatic maturation in iPS-derived hepatic cells. LHX2 overexpression in human iPS-HSCs significantly enhanced the expression of laminin family genes, such as *LAMA4*, *LAMA2*, and *LAMA5*, and decreased expression of collagen family genes (Fig. 5). A previous study reported that LHX2 overexpression in the LX-2 cell line decreased the expression of *ACTA2*; however, changes in the ECM expression profile induced by LHX2 were unclear^21^. Our analyses here revealed that the ECM expression profile, including laminin and collagen genes, is regulated by LHX2 expression in human iPS-HSCs. Consistent with our data, laminin-411, which is composed of LAMA4, LAMB1, and LAMC1, plays an essential role in the maturation and proliferation of hepatocytes and iPS-derived hepatic cells^39^. Other studies have shown that culture of iPS-derived cells on laminin-coated dishes, such as on laminin-511 and laminin-111, supports cell differentiation into the hepatic lineage and the maintenance of hepatoblast-like cells^37,38^. Our results here showed that culture on defined laminin-coated dishes induced a lower rate of hepatic maturation in iPS-HPCs compared to co-culture with iPS-HSCs (Fig. 4 and Supplementary Fig. 5f). This finding supported our conclusion on the significance of the ECM profile, which is regulated by LHX2 expression, of human iPS-HSCs for maturation of human iPS-HPCs.

In this study, iPS-HSCs were differentiated via mesodermal cells from pluripotent stem cells (Fig. 1), in a similar manner as previously reported for iPS-hepatic stellate cell-like cells^13–15^. On the other hand, several supplemented factors for induction of HSCs from iPS cells were different from those in previous reports, and we did not purify a defined fraction in differentiated cells using FACS. Vitamin A storage (judged from autofluorescence) was detected in 20% of iPS-HSCs (Fig. 2d). The expression of *ACTA2*, *COL1A1*, and *LOX* in iPS-HSCs was increased by TGF-β stimulation (Fig. 2e). These data showed that the phenotype of the iPS-HSCs used in this study was similar to that of iPS-derived hepatic stellate cell-like cells reported previously^13,14^. Furthermore, iPS-HSCs facilitates the maturation of iPS-HPCs in 2D direct co-culture, but not in transwell co-culture (Fig. 2 and Supplementary Fig. 2). Consistent with our data, murine integrin α8^+^ fetal HSCs support hepatic maturation of hepatoblast in direct co-culture, but not in transwell co-cutlure^40^, indicating that a direct cell-cell interaction between HSCs and HPCs is important for hepatic maturation of progenitors. Previous transcriptome analyses showed that iPS-derived hepatic stellate cell-like cells resembled human primary HSCs^14^. Our data and those from other studies suggest that the phenotypes of iPS-HSCs partially overlap those of primary HSCs.

The results obtained here showed that LHX2 overexpression in iPS-HSCs increased the level of expression of genes related to HSC quiescence, such as *NGFR*, *Neurotrimin*, and *LRAT*, whereas expression of genes related to HSC activation (*COL1A1*, *ACTA2*, and *LOX*) was not down-regulated by LHX2 overexpression. This suggests that LHX2 overexpression in iPS-HSCs partly contributes to the maintenance of quiescence. *Lhx2*-deficient mice develop progressive liver fibrosis during fetal development, indicating that LHX2 expression inhibits myofibroblastic changes in HSCs in mice^21^. However, *Lhx2* overexpression in mouse primary HSCs and human LX-2 cell line did not maintain the quiescence of HSCs *in vitro*^33^. One possibility for the discrepancy between our data and previous studies is that the overexpression approach influenced the final expression levels of LHX2 in host cells. Another possibility is that there are differences in the basal expression level of the *LHX2* gene in host cells. *LHX2* expression in iPS-HSCs was higher than in iPS cells, but lower than in LX-2 cells (Supplementary Fig. 3a). Thus, *LHX2* overexpression in iPS-HSCs may be effective for the maintenance of the quiescence phenotype of these cells.

The genes targeted by the transcription factor activity of LHX2 in primary HSCs remain unclear. LHX2 expression plays an important role in the differentiation of human iPS cells into hematopoietic progenitor cells; however, the mechanism of LHX2 activity during hematopoietic differentiation has not been fully elucidated^27,32^. Co-culture of a bone marrow stroma cell line overexpressing LHX2 with iPS cells enhances the hematopoietic differentiation of the iPS cells via the upregulation of the humoral factor apelin^26^. However, expression of *apelin* was not increased in Dox-treated iLHX2-HSCs compared to control cells (data not shown), indicating that apelin is not a target molecule of LHX2 in iPS-HSCs. LHX2 regulates the Wnt signaling pathway in neural development and in the maintenance of hair follicle stem cells^24,25,41^. We examined the potential role of the Wnt signaling pathway in iLHX2-HSCs; a TOP/FOP flash reporter assay revealed that LHX2-overexpression did not stimulate Wnt signaling pathway in iPS-HSCs (data not shown). The molecular mechanism regulating the expression of genes related to quiescence and to ECM production in iPS-HSCs by LHX2 expression remains unclear, and further study will be needed to address this question.

Our data demonstrated that genetically modified iPS-HSCs are a useful tool for improvement of hepatic maturation of iPS-derived hepatic cells. Several reports showed that co-culture of iPS-derived hepatic cells, stellate cells, and sinusoidal endothelial cells can recapitulate organ development and the pathological development observed in genetic and non-genetic diseases^7,14,15^. The use of patient-derived iPS-HSCs for the co-culture may offer new insights into the pathophysiology of genetic and non-genetic liver diseases whose molecular mechanism is unknown. Genetically modified iPS-HSCs cells will be helpful for validation analysis of potential mechanisms, and be valuable for further screening of novel molecular targets. Our study into the use of genetically modified iPS-HSCs will contribute to strategies using iPS cells and to future developments of new treatments for liver fibrosis as well as improvement of hepatic maturation of iPS-derived hepatic cells.

## Materials and Methods

Materials are listed in the supplementary information.

### Cell culture of iPS cells and iPS-HPCs

The human iPS cell line RIKEN2F was established from Japanese male umbilical cord fibroblasts and reprogrammed by retroviral expression of *Oct3/4*, *Klf4 Sox2*, and *c-Myc* (OKSM). The human iPS cell line PB001 was established from peripheral blood cells and reprogrammed using a Sendai virus vector expressing OKSM, and was transduced by a lentiviral vector expressing tdTOMATO. RIKEN2F and PB001 were gifted by Dr. Y. Nakamura (RIKEN)^42^ and Dr. H. Masaki (Institute of Medical Science, the University of Tokyo), respectively. Human iPS cell lines were maintained on mitomycin C-treated mouse embryonic fibroblast (MEF) feeder cells in iPS Medium (DMEM/F12 supplemented with 20% Knockout^TM^ serum replacement, 1× non-essential amino acids, 2 mM L-glutamine, and 55 μM 2-mercaptoethanol), or cultured using the Cellartis^®^ DEF-CS™ 500 culture system (Takara Bio, Shiga, Japan) according to the manufacturer’s protocol.

LX-2, a human hepatic stellate cell line, was purchased from Millipore (Billerica, MA, USA). LX-2 cells were cultured in DMEM supplemented with 10% FBS and 1× antibiotic-antimycotic mixed solution.

### Establishment of human iPS cell lines overexpressing LHX2 (iLHX2-iPS cells)

As previously described, we constructed a self-contained, tetracycline-inducible expression vector based on the PiggyBac transposon^6,31,43^. Activation of gene expression in response to Dox can be indirectly monitored by co-incident GFP production. Briefly, we produced a derivative vector expressing human *LHX2* using the Gateway cloning technique. The vector, PB-T-LHX2-G-ERN (Fig. 3a), was transfected together with PiggyBac transposase into the RIKEN-2F iPS cell line and was selected using media containing G418 for 3 days to generate pooled iPS cell lines containing genomic transposon integrations (iLHX2-iPS cells). We obtained 11 iLHX2-iPS cell lines and 4 cell lines were analyzed in this study after the evaluation of LHX2 expression levels. The resultant 2F iLHX2-iPS cell lines were differentiated into the HSC-lineage as described below.

### Differentiation of human iPS-HSCs

Human iPS cells were seeded on 12 or 24 well plates at a density of ~6 × 10^4^ cells/cm^2^. The cells were incubated in DMEM/F12 supplemented with 2% B-27 supplement (B27), 1% N-2 supplement (N2), 10 μM CHIR99012, 10 ng/ml recombinant human bFGF, 30 ng/ml recombinant human BMP-4, and 10 ng/ml recombinant activin A from days 1 to 3. Next, the cells were cultured in DMEM/F12 supplemented with 2% B27, 1% N2, 100 ng/ml bFGF and 50 ng/ml BMP-4 from days 4 to 6. Medium was changed daily from days 1 to 6. Harvested cells at day 6 were termed iPS-HSCs, and were passaged on fibronectin-coated plates and cultured in RPMI 1640 supplemented with 2% B27 for further experiments.

### Myofibroblast-like change of iPS-HSCs

Myofibroblast-like changes in iPS-HSCs were investigated using cells passaged on fibronectin-coated 24-well plates at a density of 2.5 × 10^4^ cells/cm^2^. The cells were incubated in RPMI 1640 supplemented with 2% B27 and 3 ng/ml recombinant human TGF-β1 for 4 days and then analyzed.

### Retinol storage in iPS-HSCs

Vitamin A (retinol) storage in iPS-HSCs was analyzed in cells passaged on fibronectin-coated plates or fibronectin-coated microscope slides. The cells were cultured in RPMI 1640 supplemented with 2% B27, 5 μM retinol, and 100 μM palmitic acid for 4 days. Harvested cells were stained with BODIPY493/503, and were analyzed using an FV10i confocal laser microscope (Olympus, Tokyo, Japan). For flow cytometric analysis, cells were dissociated using 0.05% trypsin/0.5 mM ethylenediaminetetraacetic acid (EDTA). The autofluorescence of harvested cells was analyzed by nUV laser (375 nm) and 440/50 filter using a FACS Aria2 cell sorter (Becton Dickinson, Franklin Lakes, NJ, USA)^44^.

### Co-culture of iPS-HPCs and iPS-HSCs

For transwell co-culture of iPS-HPCs and iPS-HSCs or iLHX2-derived HSCs (iLHX2-HSCs), or LX-2 as a control, iPS-HPCs were seeded onto fibronectin-coated culture inserts (Millipore) at a density of 5.0 × 10^4^ cells/cm^2^, and iPS-HSCs, iLHX2-HSCs, or LX-2 cells were seeded on fibronectin-coated plates at a density of 5.0 × 10^4^ cells/cm^2^. Cells were incubated in HPC medium for 1 day, and incubated in Hepatocyte Culture Medium without EGF (Lonza, Basel, Switzerland) for 6 days and then harvested. The medium was changed every other day.

For 2D direct co-culture, the CytoSelect^TM^ 24-well Cell Co-culture System (Cell Biolabs, San Diego, CA, USA) was used following the manufacturer’s protocol with slight modifications. Briefly, iPS-HSCs or iLHX2-HSCs were dissociated using 0.05% trypsin/0.5 mM EDTA, and 2.0 × 10^5^ cells were seeded into fibronectin-coated wells. The next day, 1.0 × 10^5^ tdTOMATO-labeled iPS-HPCs derived from the PB001 cell line were seeded into the iPS-HSC-seeded wells, and incubated with HPC medium for 1 day. Both cells were cultured in Hepatocyte Culture Medium without EGF for 6 days, and the medium was changed every other day. Co-cultured cells were dissociated using 0.05% trypsin/0.5 mM EDTA and were sorted using FACS. Sorted cells were then analyzed.

### Statistics

GraphPad Prism software (GraphPad Software, San Diego, CA, USA) was used to calculate the standard deviation (SD) and statistical significance by Student’s two-tailed t test between two groups or by one-way ANOVA followed by Tukey’s test between more than three groups; *P* values < 0.05 were considered statistically significant. In all graphs, bars represent the mean ± SD of three or four separate experiments.

Materials, methods for establishment of human iPS-HPCs, expression analysis using quantitative RT-PCR and microarray, flow cytometric analysis and cell sorting, immunohistochemistry, immunoblot analysis, enzyme-linked immunosorbent assay (ELISA), and knockdown assay of LHX2 using lentiviral overexpression of shRNA are shown in the Supplementary information.

## Supplementary information

Supplementary Information
