# Supplementary material for: LIM homeobox 2 promotes interaction between human iPS-derived hepatic progenitors and iPS-derived hepatic stellate-like cells
Source: Sci Rep. 2019 Feb 14;9:2072. doi: 10.1038/s41598-018-37430-9 (PMC6376133; doi:10.1038/s41598-018-37430-9)
Supplement: Supplementary file 1 — Supplementary Information [file 41598_2018_37430_MOESM1_ESM.pdf]

Supplementary Information

LIM homeobox 2 promotes interaction between human iPS-derived hepatic progenitors and iPS-derived hepatic stellate-like cells

Masato Miyoshi[1,a], Sei Kakinuma[1,2,a], Akihide Kamiya[3], Tomoyuki Tsunoda[1], Jun Tsuchiya[1], Ayako Sato[1], Shun Kaneko[1], Sayuri Nitta[1], Fukiko Kawai-Kitahata[1], Miyako Murakawa[1], Yasuhiro Itsui[1], Mina Nakagawa[1], Seishin Azuma[1], Hiromitsu Nakauchi[4,5], Yasuhiro Asahina[1,2], & Mamoru Watanabe[1]

1 Department of Gastroenterology and Hepatology, Tokyo Medical and Dental University, Tokyo, Japan.
2 Department of Liver Disease Control, Tokyo Medical and Dental University, Tokyo, Japan.
3 Department of Molecular Life Sciences, School of Medicine, Tokai University, Isehara, Japan.
4 Institute for Stem Cell Biology and Regenerative Medicine, Stanford University School of Medicine, Stanford, CA, USA.
5 Division of Stem Cell Therapy, Institute of Medical Science, The University of Tokyo, Tokyo, Japan.
a These authors contributed equally to this work.

Table of contents

Supplementary Materials and Methods	Page 2-7
Supplementary Table 1	Page 8-10
Supplementary Table 2	Page 11-12
Supplementary Figures	
Supplementary Figure 1	Page 13-14
Supplementary Figure 2	Page 15
Supplementary Figure 3	Page 16-17
Supplementary Figure 4	Page 18
Supplementary Figure 5	Page 19-20
Supplementary Figure 6	Page 21
Supplementary Figure 7	Page 22
References for Supplementary Information	Page 23

Supplementary Materials and Methods

Materials

The following reagents were used in this study: Dulbecco's modified Eagle's medium (DMEM), RPMI 1640, dexamethasone (Dex), trypsin-EDTA, 4',6-diamidine-2'-phenylindole dihydrochloride (DAPI), dimethyl sulfoxide (DMSO), retinol, and palmitic acid (Sigma, St. Louis, MO, USA); fetal calf serum (FCS) (BioWest, Nuaillé, France); DMEM/F-12, 100× insulin/transferrin/selenium (ITS), 200 mM L-Glutamine, 100× non-essential amino acid, Knockout Serum Replacement™, 2-mercaptoethanol, B-27™ supplement (50×), N-2 supplement, TrypLE™ Select (1×), BODIPY™ 493/503 (4,4-Difluoro-1,3,5,7,8-Pentamethyl-4- Bora-3a,4a-Diaza-s-Indacene), and 2.5% trypsin solution (Thermo Fisher Scientific, Waltham, MA, USA); 1 M HEPES buffer solution, and 100× antibiotic-antimycotic mixed stock solution (Nacalai Tesque, Kyoto, Japan); recombinant human basic FGF (bFGF) and iMatrix-511 (Wako Pure Chemicals, Osaka, Japan); Y27632 and nicotinamide (DS Pharma Biomedical, Osaka, Japan); A83-01 (TGF-β inhibitor) (Tocris Bioscience, Bristol, UK); recombinant human epidermal growth factor (rhEGF), recombinant human hepatocyte growth factor (rhHGF), recombinant human bone morphologic protein 4 (rhBMP-4), recombinant human activin A, recombinant human fibroblast growth factor 7 (rhFGF7), and recombinant transforming factor β-1(TGF-β1) (PeproTech, Rocky Hill, NJ, USA); CHIR99021 (ReproCELL, Kanagawa, Japan); mitomycin C (Kyowa Hakko Kirin, Tokyo, Japan); Cellartis® DEF-

CS™ 500 culture system (Takara Bio, Shiga, Japan); Hoechst 33342 (Lonza, Basel, Switzerland); fibronectin (Corning, Corning, NY, USA).

Establishment of human iPS-derived hepatic progenitor cells (iPS-HPCs)

Human iPS-HPCs were established and cultured as previously described[1,2]. Briefly, iPS cells were differentiated into the hepatic lineage using a 4-step protocol on an EHS gel-coated plate[3]. At the hepatoblast stage, the $CD13^{high}/CD133^{+}$ fraction, which includes hepatic progenitor cells of the mouse fetal liver[4], was sorted using a fluorescence-activated cell sorter (FACS), and cloned onto mouse embryonic fibroblast (MEF) feeder cells. Human iPS-HPCs were passaged and expanded in HPC medium [1:1 mixture of DEME/F12 and hepatic colony-forming units in culture (H-CFU-C) medium supplemented with 40 ng/ml recombinant human hepatocyte growth factor (rhHGF), 20 ng/ml recombinant human epidermal growth factor (rhEGF), 0.25 mM A83-01, 10 mM Y-27632, and 10% FBS]. H-CFU-C medium consisted of DMEM/F12 supplemented with 2× non-essential amino acids, 2× insulin-transferrin-selenium, 5 mM HEPES, 2×10^{-7} M dexamethasone, and 2× antibiotic-antimycotic mixed solution.

To investigate the effects of humoral factors on hepatocytic maturation, iPS-HPCs were cultured in the Hepatocyte Culture Medium without EGF (Lonza) supplemented with 10 ng/ml FGF7, 40 ng/ml HGF, and 10 ng/ml FGF7 plus 40 ng/ml HGF for 4 days. In the experiments of defined extracellular matrices, iPS-HPCs were cultured in the Hepatocyte Culture Medium without EGF on type I collagen-coated (AGC Techno Glass,

Shizuoka, Japan), fibronectin-coated, and iMatrix-511-coated dishes.

Expression analysis using quantitative reverse transcriptase-polymerase chain reaction (RT-PCR) and microarray

Total RNA was extracted using RNeasy mini or micro kits (Qiagen, Hilden, Germany) according to the manufacturer's protocols. First strand cDNA was synthesized using a Primescript™ -II 1st strand cDNA synthesis kit (Takara Bio) and used as a template for PCR amplification. Quantitative RT-PCR was carried out using the Universal Probe Library system (Roche Diagnostics, Indianapolis, IN, USA). All data was normalized against GAPDH. The primer sequences are listed in Supplementary Table 1.

For the microarray analysis, total RNA was extracted using RNeasy micro kit from iLHX2-HSC cells co-cultured with iPS-HPCs in media supplemented with vehicle or Dox after FACS sorting. A mixture of RNAs from three wells was used for the analysis. A detailed description of the methods was presented previously[5]. Briefly, we used 3D-Gene Human Oligo chip 25k (24,457 distinct genes, Toray Industries, Tokyo, Japan). Total RNA was labeled with Cy3 or Cy5 using the Amino Allyl MessageAMP II aRNA Amplification Kit (Applied Biosystems, Foster City, CA, USA). Genes with Cy3/Cy5 normalized ratios of >2.0 or <0.5 were defined as upregulated or downregulated, respectively. Pathways that showed significant gene expression changes were analyzed using GenMAPP ver2.1 (MAPP finder, http://www.genmapp.org/)

software. The full microarray data was deposited to Gene Expression Omnibus of NCBI (http://www.ncbi.nlm.nih.gov/geo/, GEO accession number, GSE122750).

Flow cytometric analysis and cell sorting

For flow cytometric analysis, cells were dissociated in 0.05% trypsin / 0.5 mM EDTA and washed twice with PBS. The cells were incubated with a primary antibody and washed with 3% FCS/PBS. The antibodies used in this study are listed in Supplementary Table 2. Incubation in the immune serum alone was used as a negative control. When unlabeled antibodies were applied, the cells were subsequently incubated with the appropriate secondary antibody. The cells were then washed in PBS and stained with propidium iodide to identify dead cells. We used BD Canto2 (Becton Dickinson, Franklin Lakes, NJ, USA) or BD Aria2 (Becton Dickinson) for the flow cytometric analysis. For cell sorting, cells were sorted using a BD Aria or BD Melody (Becton Dickinson).

Immunohistochemistry

Cultured cells were washed with PBS and fixed in 50% acetone/50% methanol. After three washes with PBS, the cells were incubated with 10% Blocking One (Nacalai Tesque)/PBS for 2 hours, and then with primary antibodies (Supplementary Table 2) overnight at 4°C. Incubation with the immune serum alone was used as a negative control. After three washes with PBS, the cells were incubated with the appropriate secondary antibody. The cells were washed with PBS and their nuclei were stained with 4’,6-

diamidino-2-phenylindole dihydrochloride (DAPI). Images were taken using a BZ-X710 microscope (Keyence, Osaka, Japan).

Immunoblot analysis and enzyme-linked immunosorbent assay (ELISA)

Immunoblot analyses were performed as previously described[5]. Briefly, cultured cells were homogenized in RIPA buffer (50 mM, pH 8.0, Tris-HCL, 150 mM NaCl, 0.1% NP-40, 1% sodium deoxycholate, 1% sodium dodecyl sulfate) containing a Halt proteinase and phosphate inhibitor cocktail (Thermo Fisher). Cell homogenates (10 µg protein) were subjected to sodium dodecyl sulfate/polyacrylamide gel electrophoresis and transferred to PVDF membranes. The blots were incubated with primary antibodies (Supplementary Table 2), followed by incubation with peroxidase-labeled secondary antibodies (GE Healthcare, Uppsala, Sweden) and visualization using the ECL Western Blotting Analysis System (GE Healthcare). Images of immunoblots were quantified using Image Lab software (Bio-Rad Laboratories, Hercules, CA, USA).

For ELISA of albumin, supernatants of cultured cells were collected and filtered through a 0.45 µm filter. Albumin protein in the supernatants was quantified using a human albumin ELISA kit (Bethyl Lab, Mongomery, TX, USA) according to the manufacturer’s instructions.

Knockdown assay of LHX2 using lentiviral overexpression of shRNA

Plasmids for lentivirus expressing shRNA targeted to human *LHX2* gene or firefly

luciferase (*Luc*, negative control) were purchased from Sigma-Aldrich (MISSION^{TM} shRNA). Lentivirus was produced in 293T cells as previously described[6]. For the 2D co-culture experiment, 2.0×10^5 LX-2 cells were seeded onto CytoSelect^{TM} 24-well Cell Co-culture System (Cell Biolabs, San Diego, CA, USA) and transduced with a lentiviral vector at a MOI 3. After 24 h transduction, the cells were washed with PBS, and 1.0×10^5 tdTOMATO-labeled iPS-HPCs derived from the PB001 cell line were seeded into the LX-2-seeded wells, and incubated with HPC medium for 1 day. Both cells were cultured in Hepatocyte Culture Medium without EGF for 6 days, and the medium was changed every other day. Co-cultured cells were dissociated using 0.05% trypsin/0.5 mM EDTA and were sorted using FACS. Sorted cells were then analyzed.

Supplementary Tables

Supplementary Table 1. Primers used for quantitative reverse transcriptase-polymerase chain reaction

Gene	Forward sequence	Reverse Sequence	UPL Probe#
**GAPDH**	AGCCACATCGCTCAGACAC	GCCCAATACGACCAAATCC	60
**OCT3/4**	GAAACCCACACTGCAGATCA	CGGTTACAGAACCACACTCG	3
**Brachyury**	GCTCACCAATGAGATGATCG	AGACACGTTCACCTTCAGCA	51
**MESP1**	GAAGGGCAGGCGATGGAG	ACCACTTCGAAGGTGCTGAG	27
**FOXF1**	CAGCCTCTCCACGCACTC	CCTTTCGGTCACACATGCT	5
**LHX2**	CCAAGGACTTGAAGCAGCTC	AAGAGGTTGCGCCTGAACT	85
**WT1**	AGCTCAAAAGACACCAAAGGAG	GGGAGAACTTTCGCTGACAA	47
**ALCAM**	CAGTTCCTGCCGTCTGCT	CTGAATTTACAGTATACCATCCA	34
**NGFR**	TCATCCCTGTCTATTGCTCCA	TGTTCTGCTTGCAGCTGTTC	66
**HGF**	CAGCATGTCCTCCTGCATC	TCTTTTCCTTTGTCCCTCTGC	15
**CYGB**	CCGCTGCCTACAAGGAAGT	GGGTGGAGTTAGGGGTCCT	62
**LRAT**	AAAGCTGCTGGGCTTTACC	ATTATCTTCACAGTCTGACAAAA	40
**PDGFRα**	CCACCTGAGTGAGATTGTGG	TCTTCAGGAAGTCCAGGTGAA	27
**LOX**	GGGAATGGCACAGTTGTCA	ACTTGCTTTGTGGCCTTCAG	82

**ACTA2**	CACCCAGCACCATGAAGAT	CGTATTCCTGTTTGCTGATCC	11
**COL1A1**	CCCCTGGAAAGAATGGAGAT	AATCCTCGAGCACCCTGA	60
**AFP**	ATGGCCATCACCAGAAAAAT	CATAAGTGTCCGATAATAATGTCAGC	66
**ALB**	AATGTTGCCAAGCTGCTGA	CTTCCCTTCATCCCGAAGTT	27
**CYP7A1**	GCAGGCACCTGTAGTCTTAGC	CGGAGACGGGATCTCACTA	64
**APOB**	GACGACTTTTCTAAATGGAACTTCTAC	CTCAGTTTTGAATATGGTGAGTTTTT	55
**Neurotorimin**	GCCTACGGTTACTTGGAGACA	GGAGGCACTGCACTCGTAG	33
**Desmin**	ACAACCTGCTCGACGACCT	TTCTCTGCTTCTTCCTTCAACTG	63
**COL1A2**	TCTGGAGAGGCTGGTACTGC	CCAGGGAGACCCAGAATACC	85
**COL4A5**	CAGGACCAAAGGGTAATCCA	GACCAATTGGCCCTGGTAT	85
**COL5A1**	AGCGTGGGAAACTGCTCTC	TGGTAGGTGACGTTCTGGTG	66
**COL3A1**	CTGGACCCCAGGGTCTTC	CATCTGATCCAGGGTTTCCA	20
**LAMA2**	GCTCCCTATCTGGGAAACAA	TGATATGGTAAATGTCAACTGTCCTC	3
**LAMA5**	TGACTGAGGCCTGCACATAC	TGTAGAGGAGGCAGTTGTCG	3
**LAMA4**	GGATGCCGAAGACATGAAC	TTCCCTCACTCTTTCCTGTTGT	66
**PPARγ**	GACAGGAAAGACAACAGACAAATC	GGGGTGATGTGTTTGAACTTG	7

GAPDH, glyceraldehyde-3-phosphate dehydrogenase; MESP1, mesoderm posterior basic helix-loop-helix transcription factor 1; FOXF1, forkhead box F1; LHX2, LIM homeobox 2; WT1, Wilms tumor 1; ALCAM, activated leukocyte cell adhesion molecule; NGFR, nerve growth factor receptor; HGF, hepatocyte growth factor; CYGB, cytoglobin; LRAT, lecithin retinol acyltransferase; PDGFRα, platelet-derived growth

factor receptor; LOX, lysyl oxidase; ACTA2, actin, alpha 2, smooth muscle, aorta; COL1A1, collagen type I alpha 1 chain; AFP, α-fetoprotein; ALB, albumin; CYP7A1, cytochrome P450 family 7 subfamily A member 1; APOB, apolipoprotein B; COL1A2, collagen type I alpha 2 chain; COL4A5; collagen type IV alpha 5 chain; COL5A1, collagen type V alpha 1 chain; COL3A1, collagen type III alpha 1 chain; LAMA2, laminin subunit alpha 2; LAMA5, laminin subunit alpha 5; LAMA4, laminin subunit alpha 4; PPARγ, peroxisome proliferator-activated receptor gamma.

Supplementary Table 2. Antibodies used in immunostaining, flow cytometry, and immunoblotting

**Antibody**	**Experiment**	**Dilution**	**Source (clone) & catalog number**
Desmin	IHC	1:100	Santa Cruz, sc-14026
AlexaFlour-488 donkey anti-rabbit IgG	IHC	1:1000	Thermo Fisher
PDGF receptor α (D13C6) XP® Rabbit mAb	FCM	1:1000	Cell Signaling Technology, #5241
FITC anti-human CD271 (NGFR) antibody	FCM	1:1000	BioLegend (ME20.4), #345103
Alexa Fluor 647 anti-human CD309 (VEGFR2/FLK-1) antibody	FCM	1:1000	BioLegend (7D4-6), #359909
PE/Cy7 anti-human CD56 (NCAM) antibody	FCM	1:1000	BioLegend (HCD56), #318317
Alexa Flour 488 anti-human CD326 (EpCAM) antibody	FCM	1:1000	BioLegend (9C4), #324209
β-actin	IB	1:5000	Sigma, A5441
LHX2	IB	1:1000	Millipore, ABE1402
ECL™ anti-mouse IgG horseradish peroxidase linked whole antibody	IB	1:2000	GE Healthcare, NA931V
ECL™ anti-rabbit IgG horseradish peroxidase linked whole antibody	IB	1:2000	GE Healthcare, NA934V

IHC, immunohistochemistry; FCM, flowcytometry; IB, immunoblot; PDGF Receptor α, platelet-derived growth factor receptor alpha; NGFR, nerve growth factor receptor; VEGFR2 / FLK-1, Vascular endothelial growth factor receptor 2 / fetal liver kinase-1; NCAM, neural cell adhesion molecule; EpCAM, epithelial cell adhesion molecule; LHX2, LIM homeobox 2.

Supplementary Figures

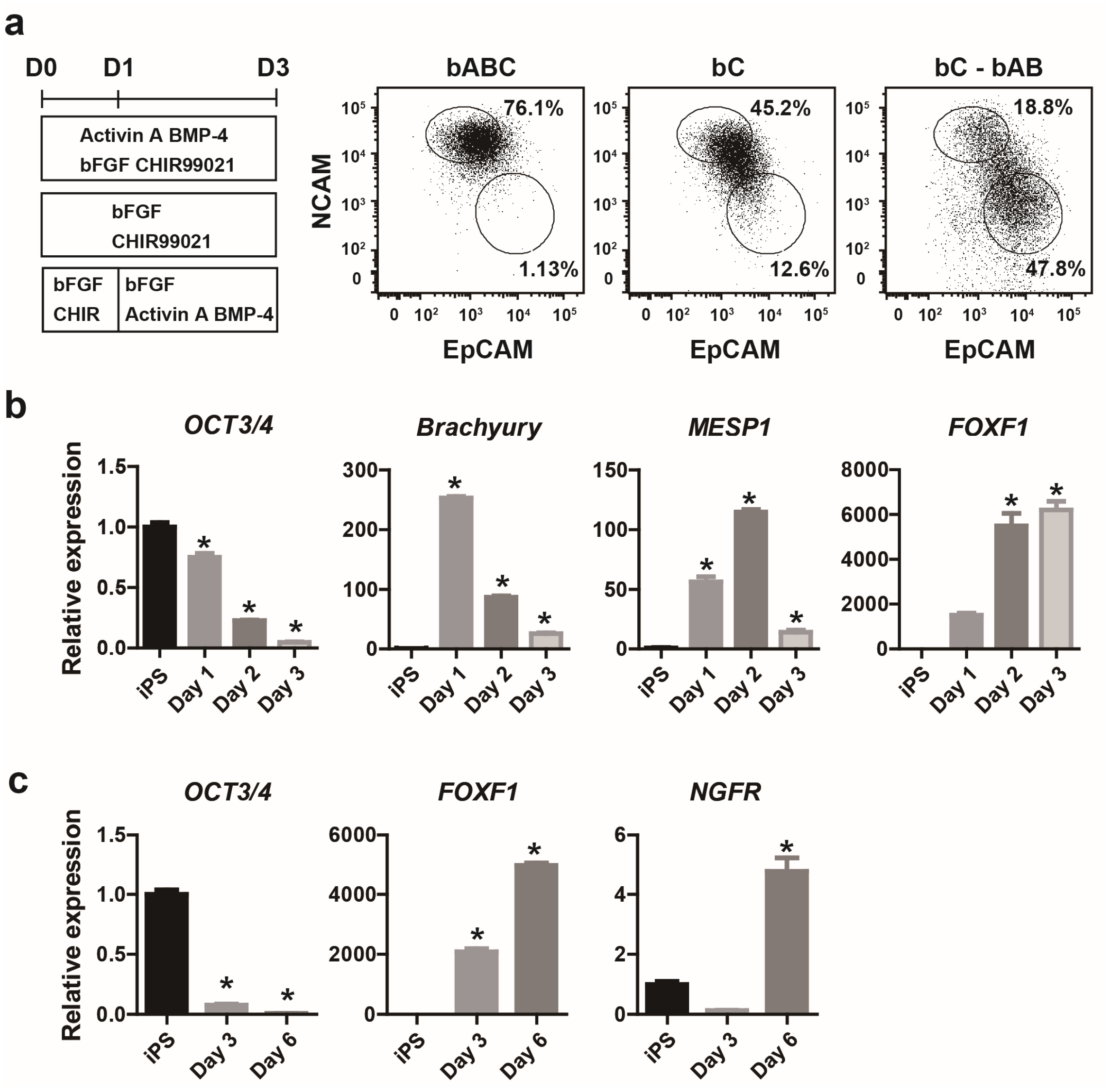

Supplementary Figure 1. Differentiation of human iPS-derived mesodermal progenitor (iPS-MP) cells and hepatic stellate cell-like cells (iPS-HSCs).

(a) Left panel: protocols for differentiation of human iPS-derived mesodermal progenitor cells differentiation. bABC protocol: 10 ng/ml bFGF, 10 μM CHIR99021, 30 ng/ml bone morphogenetic protein-4 (BMP-4), and 10 ng/ml activin A. bC protocol: 10 ng/ml basic fibroblast growth factor (bFGF), and 10 μM CHIR99021. bC-bAB protocol: Day 1, 10 ng/ml bFGF and 10 μM CHIR99021. Days 2–3, 10 ng/ml bFGF and 30 ng/ml BMP-4, and 10 ng/ml activin A. Right

panel: Flow cytometric analysis of cells after incubation for 3 days. The x- and y-axes represent epithelial cell adhesion molecule (EpCAM) and neural cell adhesion molecule (NCAM), respectively. The $NCAM^{+}/EpCAM^{-}$ fraction in iPS-derived cells are iPS-MP cells. (b) Expression of *OCT3/4*, *Brachyury*, mesoderm posterior basic helix-loop-helix transcription factor 1 (*MESP1*), and forkhead box F1 (*FOXF1*) in human iPS-derived cells (RIKEN-2F) at day 0 (iPS cells), day 1, day 2, and day 3 cultured using the bABC protocol. The y-axis represents the ratio of expression relative to means of day 0 (iPS cells). (c) Differentiation of another iPS cell line, PB001, into iPS-HSC cultured using the protocol shown in Fig.1a. Expression of *OCT3/4*, *FOXF1*, and nerve growth factor receptor (*NGFR*) are shown. The y-axis represents the ratio of expression relative to means of day 0 (iPS cells). Results represent the mean ± SD of three separate experiments. $*P < 0.05$.

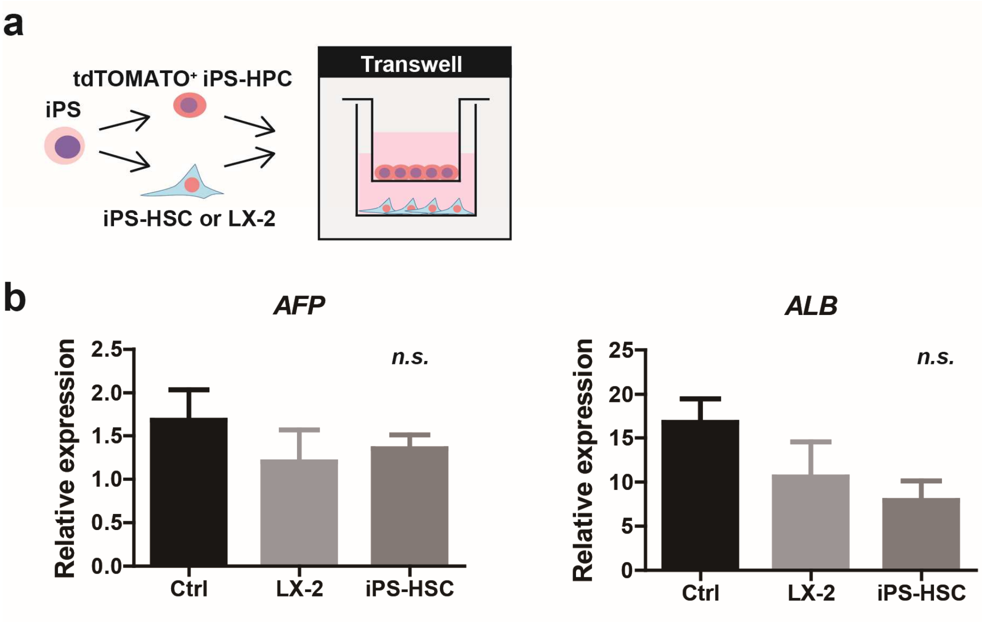

Supplementary Figure 2. Transwell co-culture of iPS-HSCs and human iPS-derived hepatic progenitor cells (iPS-HPCs).

(a) Schema for the transwell co-culture of tdTOMATO$^+$ iPS-HPCs (red) and iPS-HSCs using cell culture inserts. (b) Expression of α-fetoprotein (*AFP*) and albumin (*ALB*) in iPS-HPCs co-cultured with LX-2 cells or iPS-HSCs. The y-axis represents the ratio of expression relative to the mean of iPS-HPCs cultured alone (control, Ctrl). Results represent the mean ± SD of three separate experiments. *$P < 0.05$.

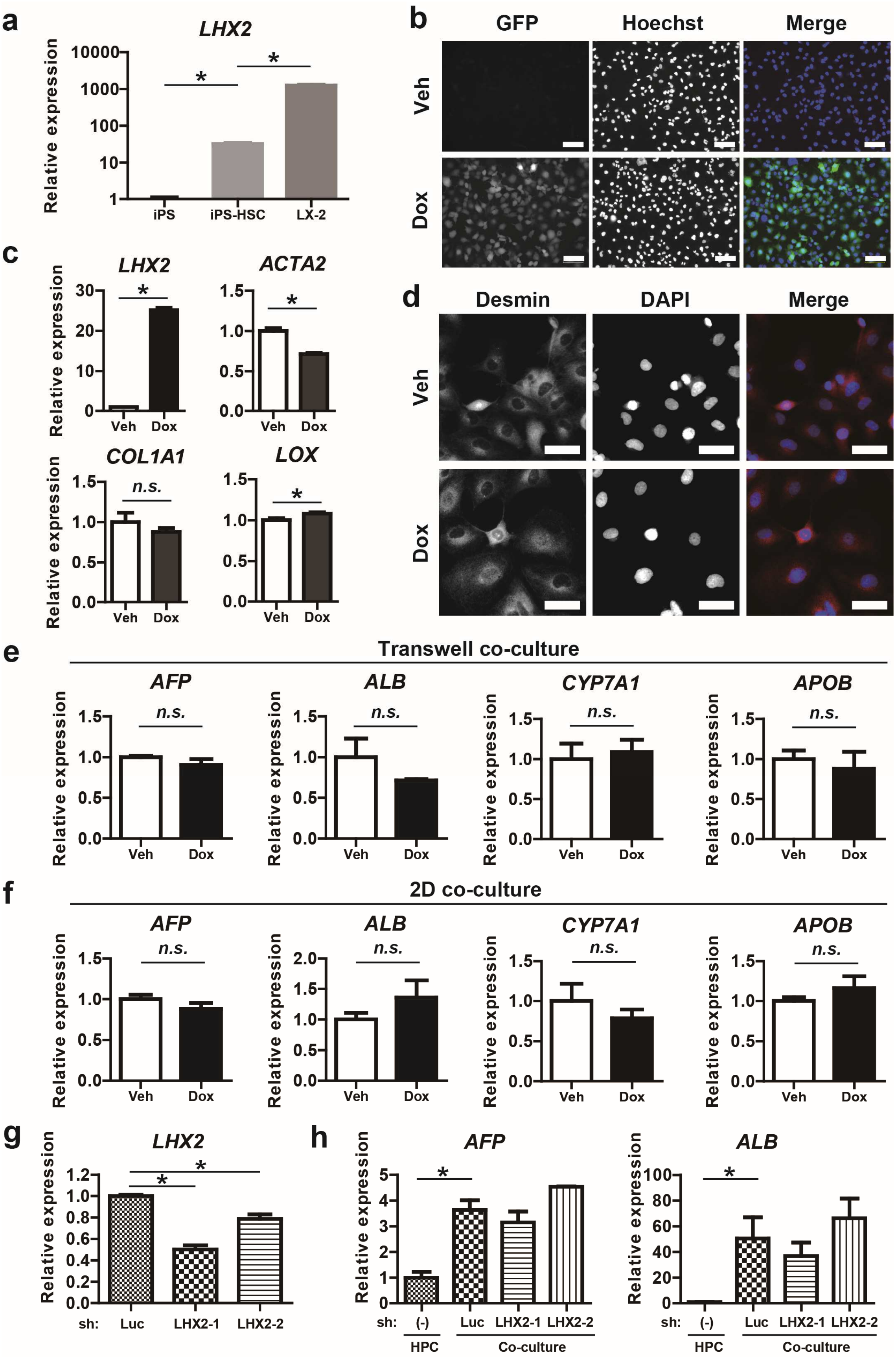
a
LHX2
Relative expression
10000
1000
100
10
1
*
*
iPS
iPS-HSC
LX-2
b
GFP
Hoechst
Merge
Veh
Dox
c
LHX2
ACTA2
COL1A1
LOX
Relative expression
Veh Dox
n.s.
d
Desmin
DAPI
Merge
Veh
Dox
e
Transwell co-culture
AFP
ALB
CYP7A1
APOB
n.s.
Relative expression
Veh
Dox
f
2D co-culture
AFP
ALB
CYP7A1
APOB
n.s.
g
LHX2
sh:
Luc
LHX2-1
LHX2-2
h
AFP
ALB
(-)
HPC
Co-culture

Supplementary Figure 3. Overexpression and silencing of LHX2 in a human hepatic stellate cell line, LX-2.

(a) Expression levels of *LHX2* in iPS cells, iPS-HSCs and LX-2 cells. The y-axis represents the ratio of expression relative to iPS cells. (b) Expression of green fluorescent protein (GFP) in LX-2-LHX2 (tetracyclin-inducible LHX2 overexpression) treated with doxycycline (Dox) or vehicle (Veh). Nuclei were stained with Hoechst 33342 (blue). Scale bars: 100 μm. (c) Expression of *LHX2*, α-smooth muscle actin (*ACTA2*), type I collagen, alpha 1 chain (*COL1A1*), and lysyl oxidase (*LOX*) in Veh- or Dox-treated LX-2-LHX2 cells was analyzed using quantitative RT-PCR. The y-axis represents the ratio of expression relative to Veh-treated LX-2-LHX2 cells. (d) Immunostaining of desmin (red) in LX-2-LHX2 treated with Veh or Dox. Nuclei were stained with 4',6-diamidine-2'-phenylindole dihydrochloride (DAPI, blue). Scale bars: 50 μm. (e) Expression of *AFP*, *ALB*, cytochrome P450, family 7, subfamily A, polypeptide 1 *(CYP7A1)*, and apolipoprotein B (*APOB*) in iPS-HPCs in transwell co-culture with Veh- or Dox-treated LX-2-LHX2 cells. (f) Expression of *AFP*, *ALB*, *CYP7A1*, and *APOB* in iPS-HPCs in 2D direct co-culture with Veh- or Dox-treated LX-2-LHX2 cells. The y-axis represents the ratio of expression relative to the means of iPS-HPCs with vehicle-treated LX-2-LHX2 cells. (g) Knockdown of LHX2 in LX-2 cells. Expression of *LHX2* in LX-2 cells infected with lentiviruses expressing shRNAs (shLHX2-1 or shLHX2-2) was shown. LX-2 cells infected with lentivirus expressing shRNAs against luciferase (shLuc) were used as a control. The y-axis represents the ratio of expression relative to the means of LX-2 infected with lentivirus expressing shLuc. Knockdown efficiency of shLHX2-1 and shLHX2-2 was approximately 50% and 20%, respectively. (h) Expression of *AFP* and *ALB* in iPS-HPCs in 2D direct co-culture with LX-2 infected with lentiviruses. The y-axis represents the ratio of expression relative to iPS-HPCs cultured without LX-2. Expression of *AFP* and *ALB* in iPS-HPCs co-cultured with LX-2-shLuc was significantly up-regulated relative to iPS-HPCs without co-culture, validating the hepatocytic maturation of iPS-HPCs by co-culture. Results represent the mean ± SD of three separate experiments. $*P < 0.05$.

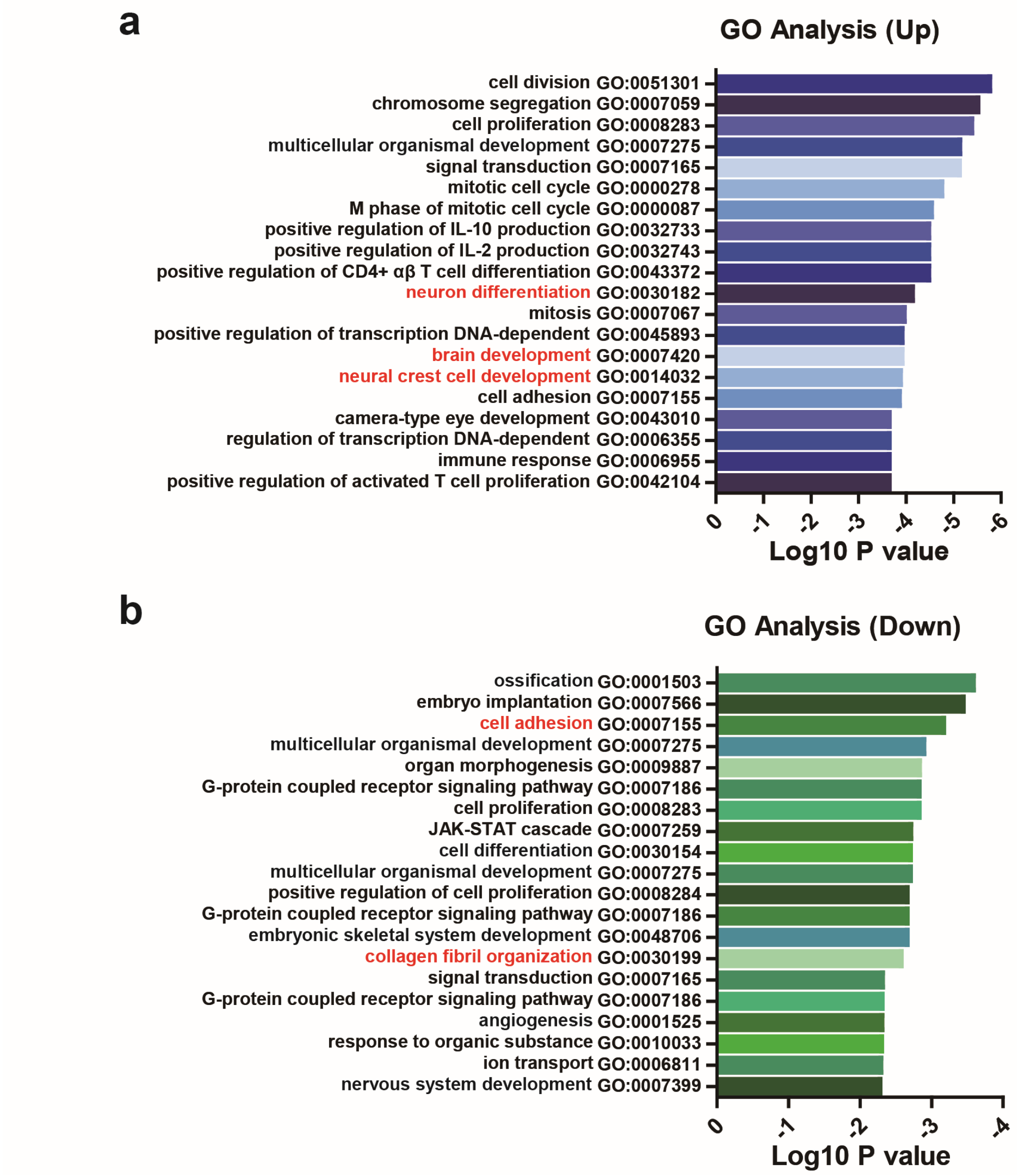

Supplementary Figure 4. Analysis of signaling pathways regulated by LHX2 overexpression in iLHX2-HSCs based on microarray analysis.

Up-regulated (a) and down-regulated (b) signaling pathways in Dox-treated iLHX2-HSCs co-cultured with iPS-HPCs relative to Veh-treated iLHX2-HSCs co-cultured with iPS-HPCs are shown; these are based on the microarray data. The y-axis represents the pathway category and gene ontogeny (GO) number. The x-axis represents log_{10} P value of each pathway.

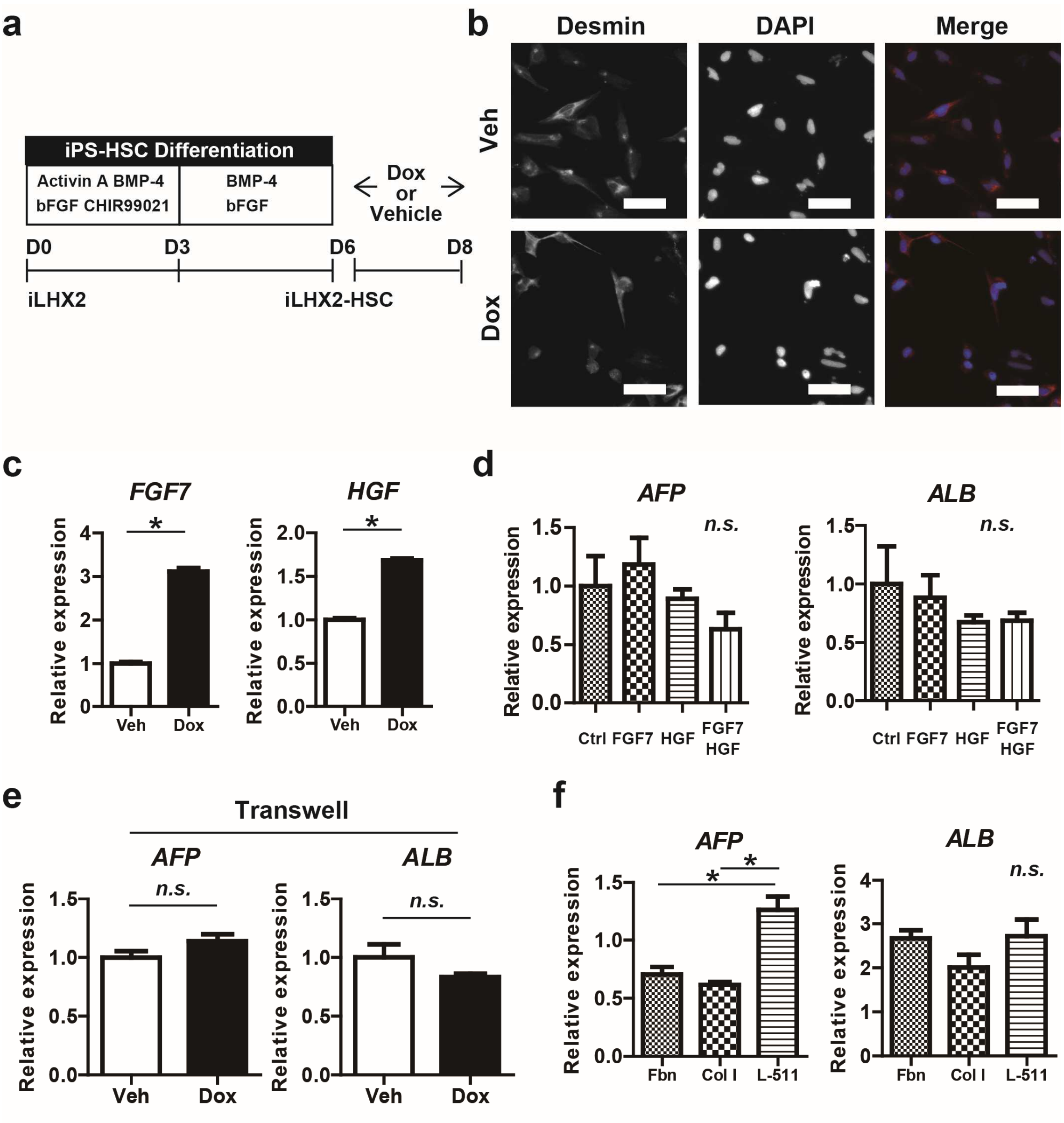

Supplementary Figure 5. Humoral factors are not responsible for hepatic maturation of iPS-HPCs induced by Dox-treated iLHX2-HSCs.

(a) Schema for experiment of LHX2 overexpression in iLHX2-HSCs. (b) Immunostaining of desmin (red) in iLHX2-HSCs treated with Veh or Dox. Nuclei were stained with DAPI (blue). Scale bars: 50 µm. (c) Expression of fibroblast growth factor 7 (*FGF7*) and hepatocyte growth factor (*HGF*) in iLHX2-HSCs treated with vehicle (Veh) or Dox. (d) Expression analysis of *AFP* and *ALB* in iPS-HPCs treated with FGF7 and/or HGF. PB001-derived iPS-HPCs were incubated with “Hepatocyte Culture Media without EGF (Lonza)” supplemented with 10 ng/ml FGF7 and/or 40 ng/ml HGF for 4 days. The y-axis represents the ratio of expression relative to Veh-

treated control (Ctrl). (e) Transwell co-culture of iPS-HPCs and iLHX2-HSCs treated with Veh or Dox. Expression of *AFP* and *ALB* in iPS-HPCs was not changed by LHX2 expression. The y-axis represents the ratio of expression relative to the means of iPS-HPCs before co-culture. (f) PB001-derived iPS-HPCs were cultured in “Hepatocyte Culture Medium without EGF” on fibronectin (Fbn)-, type I collagen (Col I)-, or iMatrix-511(laminin-511, L-511)-coated plates for 4 days. Expression of *AFP* and *ALB* were analyzed. The y-axis represents the ratio of expression relative to the means of iPS-HPCs before co-culture. Results represent the mean ± SD of three separate experiments. *$P < 0.05$.

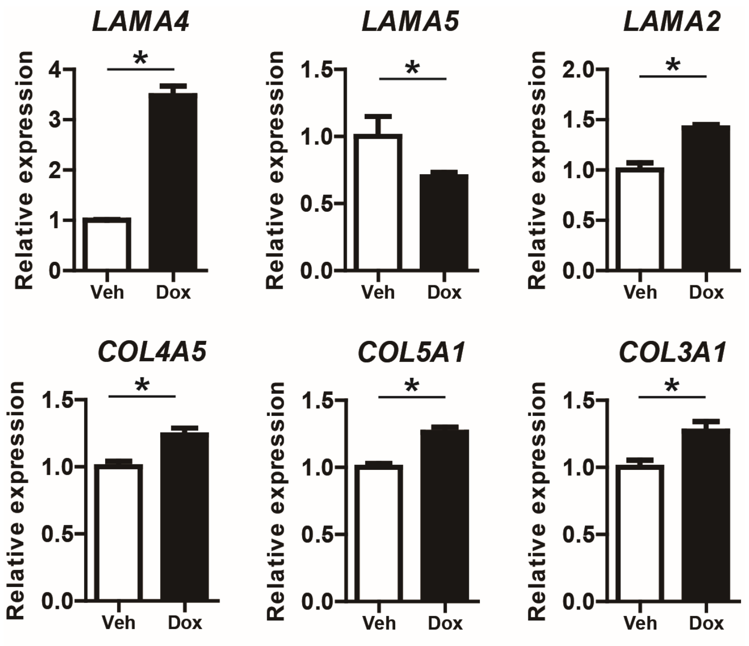

Supplementary Figure 6. Expression of extracellular matrices in LX-2-LHX2.

Quantitative RT-PCR analysis of laminin (*LAMA2*, *LAMA4*, and *LAMA5*) and collagen (*COL3A1*, *COL4A5*, and *COL5A1*) in LX-2-LHX2 cultured for 2 days with Veh or Dox. The y-axis represents the ratio of expression relative to Veh-treated LX-2-LHX2. Results represent the mean ± SD of three separate experiments. *$P < 0.05$.

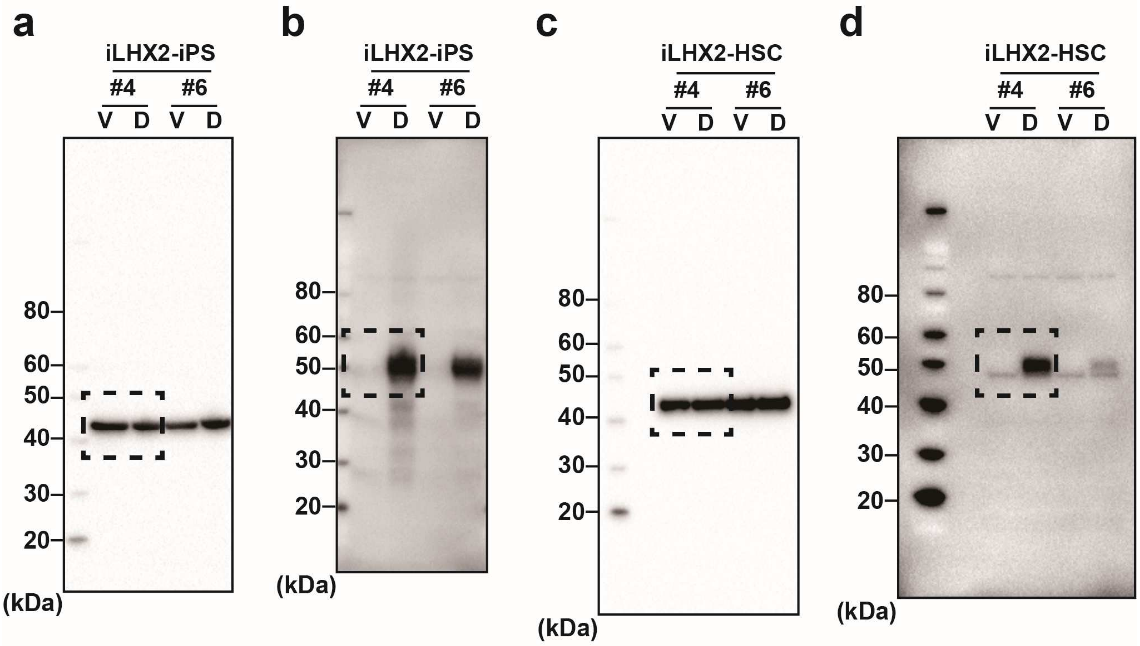

Supplementary Figure 7. Full-length gels and blots before cropping for Figure 3d.

(a, b) Immunoblots of clone number 4 (#4) and 6 (#6) iLHX2-iPS cells. Representative full-length gels using anti-β-actin antibody (a) and anti-LHX2 antibody (b) are shown. (c, d) Immunoblots of iLHX2-iHSCs derived from clone #4 and #6 iLHX2-iPS cells. Representative full-length gels using anti-β-actin antibody (c) and anti-LHX2 antibody (d) are shown. V and D indicate vehicle-treated cells and Dox-treated cells, respectively.

Supplementary References

1 Yanagida, A., Ito, K., Chikada, H., Nakauchi, H. & Kamiya, A. An in vitro expansion system for generation of human iPS cell-derived hepatic progenitor-like cells exhibiting a bipotent differentiation potential. *PLoS One* **8**, e67541, doi:10.1371/journal.pone.0067541 (2013).

2 Kaneko, S. *et al.* Human induced pluripotent stem cell-derived hepatic cell lines as a new model for host interaction with hepatitis B virus. *Sci Rep* **6**, 29358, doi:10.1038/srep29358 (2016).

3 Si-Tayeb, K. *et al.* Highly efficient generation of human hepatocyte-like cells from induced pluripotent stem cells. *Hepatology* **51**, 297-305, doi:10.1002/hep.23354 (2010).

4 Kakinuma, S. *et al.* Analyses of cell surface molecules on hepatic stem/progenitor cells in mouse fetal liver. *J Hepatol* **51**, 127-138, doi:10.1016/j.jhep.2009.02.033 (2009).

5 Kiyohashi, K. *et al.* Wnt5a signaling mediates biliary differentiation of fetal hepatic stem/progenitor cells in mice. *Hepatology* **57**, 2502-2513, doi:10.1002/hep.26293 (2013).

6 Otani, S. *et al.* Matrix metalloproteinase-14 mediates formation of bile ducts and hepatic maturation of fetal hepatic progenitor cells. *Biochem Biophys Res Commun* **469**, 1062-1068, doi:10.1016/j.bbrc.2015.12.105 (2016).
